# Mediation of the Same Epigenetic and Transcriptional Effect by Independent Osteoarthritis Risk–Conferring Alleles on a Shared Target Gene, *COLGALT2*

**DOI:** 10.1002/art.42427

**Published:** 2023-04-11

**Authors:** Yulia S. Kehayova, J. Mark Wilkinson, Sarah J. Rice, John Loughlin

**Affiliations:** ^1^ Biosciences Institute, Newcastle University Newcastle upon Tyne UK; ^2^ Department of Oncology and Metabolism University of Sheffield Sheffield UK

## Abstract

**Objective:**

Over 100 DNA variants have been associated with osteoarthritis (OA), including rs1046934, located within a linkage disequilibrium block encompassing part of *COLGALT2* and *TSEN15*. The present study was undertaken to determine the target gene(s) and the mechanism of action of the OA locus using human fetal cartilage, cartilage from OA and femoral neck fracture arthroplasty patients, and a chondrocyte cell model.

**Methods:**

Genotyping and methylation array data of DNA from human OA cartilage samples (n = 87) were used to determine whether the rs1046934 genotype is associated with differential DNA methylation at proximal CpGs. Results were replicated in DNA from human arthroplasty (n = 132) and fetal (n = 77) cartilage samples using pyrosequencing. Allelic expression imbalance (AEI) measured the effects of genotype on *COLGALT2* and *TSEN15* expression. Reporter gene assays and epigenetic editing determined the functional role of regions harboring differentially methylated CpGs. In silico analyses complemented these experiments.

**Results:**

Three differentially methylated CpGs residing within regulatory regions were detected in the human OA cartilage array data, and 2 of these were replicated in human arthroplasty and fetal cartilage. AEI was detected for *COLGALT2* and *TSEN15*, with associations between expression and methylation for *COLGALT2*. Reporter gene assays confirmed that the CpGs are in chondrocyte enhancers, with epigenetic editing results directly linking methylation with *COLGALT2* expression.

**Conclusion:**

*COLGALT2* is a target of this OA locus. We previously characterized another OA locus, marked by rs11583641, that independently targets *COLGALT2*. The genotype of rs1046934, like rs11583641, mediates its effect by modulating expression of *COLGALT2* via methylation changes to CpGs located in enhancers. Although the single‐nucleotide polymorphisms, CpGs, and enhancers are distinct between the 2 independent OA risk loci, their effect on *COLGALT2* is the same. *COLGALT2* is the target of independent OA risk loci sharing a common mechanism of action.

## INTRODUCTION

Genome‐wide association studies (GWAS) have identified over 100 DNA variants that are associated with osteoarthritis (OA) risk (1, 2, 3, 4). Biologic comprehension of GWAS signals requires elucidation of the molecular effects of the risk‐conferring alleles on their target genes (5, 6, 7, 8). Since the individual contribution of most variants to disease risk is small, assessing these effects is challenging (5, 6, 7, 8). Furthermore, determining the causal variants underpinning an association signal is not straightforward, as variants commonly occur within linkage disequilibrium (LD) blocks (5, 6, 7, 8). Despite these difficulties, the application of statistical fine mapping combined with laboratory‐based studies is generating functional insight into the molecular basis of OA genetic risk (3, 4, 9, 10, 11, 12, 13, 14, 15).

As with other polygenic diseases, most OA‐associated variants reside within the noncoding genome and contribute to disease by altering expression of genes within the same topologically associated domain (TAD), thereby acting as expression quantitative trait loci (eQTLs) (1). We have reported that DNA methylation (DNAm) at CpG dinucleotides is also often associated with genotype at OA‐associated variants, forming methylation QTLs (mQTLs), and that this epigenetic effect may act as an intermediate between the risk allele and the change in gene expression (16, 17, 18, 19, 20, 21).

One recent example was our investigation of the OA association signal marked by single‐nucleotide polymorphism (SNP) rs11583641 (22). This variant resides within the 3′‐untranslated region of *COLGALT2*, which encodes a galactosyltransferase that posttranslationally modifies collagen (22). We discovered that the OA risk allele of rs11583641 is associated with lower methylation levels of CpGs within an intronic enhancer of *COLGALT2* and that this reduced methylation increases enhancer activity and *COLGALT2* expression (22). Increased glycosylation of collagen reduces intermolecule crosslinking, leading to collagen fibrils with reduced diameters and lower tensile strength (23). We concluded that increased *COLGALT2* expression, and therefore increased galactosyltransferase activity, could be detrimental to cartilage health via effects on collagen biosynthesis (22). We subsequently reported that for some OA risk loci, including rs11583641, genotype associations with gene expression and CpG methylation observed in human arthroplasty cartilage are also observed in human fetal cartilage (24). This implies that OA genetic risk may be programmed during development.

A second association signal was recently reported that maps close to *COLGALT2* (4). Three SNPs were highlighted: rs12047271 and rs1327123, residing between *COLGALT2* and *TSEN15*, and rs1046934, residing within *TSEN15*. The TSEN15 protein is a subunit of a transfer RNA (tRNA)–splicing endonuclease. The splicing of introns from pre‐tRNAs is performed by a heterotetrameric endonuclease composed of TSEN15, TSEN34, TSEN2, and TSEN54 (25, 26). TSEN15 and TSEN34 are the structural subunits of the endonuclease, whereas TSEN2 and TSEN54 form the catalytic domains (26). TSEN15 adopts a compact α‐α‐β‐β‐β‐β‐α‐β‐β fold, preceded by a disordered N‐terminal region, which has not been structurally resolved (25, 26).

The 3 SNPs rs12047271, rs1327123, and rs1046934 are part of an LD block containing 21 SNPs (r^2^ ≥ 0.8 in European ancestry cohorts) spanning 30 kb. Furthermore, they are in near perfect linkage equilibrium with rs11583641 (r^2^ = 0, D′ ≤0.08). This second *COLGALT2* signal, which we henceforth refer to as the rs1046934 locus, is therefore genetically independent of the first *COLGALT2* signal. Here, we set out to investigate the gene targets of this new OA locus using a range of techniques.

## PATIENTS AND METHODS

### Protein modeling

TSEN15 crystal structures were downloaded from the Protein Data Bank (Supplementary Table 1, available on the *Arthritis & Rheumatology* website at https://onlinelibrary.wiley.com/doi/10.1002/art.42427) and visualized in complex with TSEN34 (6Z9U) and as a monomeric structure (2GW6) using the PyMOL Molecular Graphics System (Schrödinger). The PyMOL Mutagenesis Wizard was used to perform in silico mutagenesis to model the missense variant Gln^59^‐His introduced by rs1046934. We used the tools gnomAD (27), PolyPhen, and Mutation Taster (Supplementary Table 1) to predict the effects of this variant and of Gly^19^‐Asp, introduced by rs2274432, on TSEN15 function.

### Cartilage samples and ethics approval

Cartilage samples were obtained from 132 patients undergoing arthroplasty at the Newcastle upon Tyne NHS Foundation Trust hospitals for primary hip OA (n = 43), primary knee OA (n = 63), or femoral neck fracture (n = 26) (Supplementary Table 2, available on the *Arthritis & Rheumatology* website at https://onlinelibrary.wiley.com/doi/10.1002/art.42427). Ethics approval was granted by the NHS Health Research Authority, with donors providing written consent (19/LO/0389). Nucleic acids were extracted as previously described (20, 21, 22). Seventy‐seven matched fetal DNA and RNA samples (Supplementary Table 3, available at https://onlinelibrary.wiley.com/doi/10.1002/art.42427) were provided by the Human Developmental Biology Resource (project 200363) (24). Nucleic acids were extracted as previously described (24).

### Genotyping

Allelic quantification pyrosequencing assays were designed using PyroMark Assay Design (Qiagen) with oligonucleotide primers ordered from Integrated DNA Technologies (IDT). DNA encompassing the SNP of interest underwent polymerase chain reaction (PCR) amplification using the PyroMark PCR kit (Qiagen), with the genotype determined using the PyroMark Q24 Advanced System (Qiagen). Supplementary Table 4, available at https://onlinelibrary.wiley.com/doi/10.1002/art.42427, lists the oligonucleotide sequences.

### Allelic expression imbalance (AEI)

Proxy transcript SNPs (Supplementary Table 5, available at https://onlinelibrary.wiley.com/doi/10.1002/art.42427) were used to investigate rs114661926 AEI for *COLGALT2* (r^2^ = 0.79 with rs1046934) and rs2274432 AEI for *TSEN15* (r^2^ = 1 with rs1046934). Patients' compound heterozygote at rs1046934 and the respective transcript SNP were investigated. Complementary DNA (cDNA) was reverse transcribed from 500 ng RNA using SuperScript IV (Invitrogen). The relative ratio of the risk to nonrisk allele at the SNPs was quantified by pyrosequencing in DNA and cDNA, as previously described (17, 20, 22). Oligonucleotides were obtained from IDT (Supplementary Table 4, available at https://onlinelibrary.wiley.com/doi/10.1002/art.42427). Triplicate measures were performed and excluded if the difference was >5%. Allelic expression ratio in cDNA was normalized to allelic expression ratio in DNA for each patient.

### Discovery of mQTLs

Genotype and methylation data previously generated from the cartilage DNA of 87 hip or knee arthroplasty OA patients (28) were used. We tested CpGs 200 kb upstream and 200 kb downstream of rs1046934, encompassing the TAD for the association signal.

### Replication of mQTLs

CpGs with nominal *P* < 0.05 in the mQTL discovery were replicated in an independent cohort of cartilage arthroplasty samples and in fetal cartilage samples. DNAs were genotyped at rs1046934 by pyrosequencing. For methylation quantification, 500 ng of DNA was bisulfite converted using EZ DNA methylation kits (Zymo Research). The CpG regions were PCR amplified in bisulfite–converted DNA with methylation levels quantified using the PyroMark Q24 Platform (Qiagen). Duplicate measures were performed and excluded if the difference was >5%. Oligonucleotide sequences, which were generated by PyroMark Assay Design (Qiagen), were obtained from IDT (Supplementary Table 4, available at https://onlinelibrary.wiley.com/doi/10.1002/art.42427).

### In silico analysis

The public databases that we used are listed in Supplementary Table 1 (available at https://onlinelibrary.wiley.com/doi/10.1002/art.42427). The Roadmap project and the 3D and University of California Santa Cruz Genome Browser databases were searched to identify regulatory functions of the regions encompassing associated SNPs and mQTL CpGs, focusing on human musculoskeletal cells: primary mesenchymal stem cells (MSCs), MSC‐derived chondrocytes (E049) and adipocytes, adipose‐derived MSCs, and primary osteoblasts. Pairwise LD between SNPs in European ancestry cohorts was determined using LDlink. Transcription factor (TF)–binding profiles and the predicted impact of SNP alleles on TF binding was assessed using the JASPAR and SNP2TFBS databases, respectively.

To assess whether SNPs or CpGs were in open or closed chromatin, we investigated ATAC‐sequencing data generated from the cartilage chondrocytes of 5 knee OA patients and 5 hip OA patients and from 6 fetal knee and 6 fetal hip samples (24) (GEO accession no. GSE214394). Expression of TFs was assessed using RNA‐sequencing data generated from the hip cartilage of 10 OA patients and 6 femoral neck fracture arthroplasty patients (29; GEO accession no. GSE111358).

### Reporter gene assay

Regions surrounding cg15204595 (290 bp) and cg21606956 (260 bp) were cloned into the Lucia CpG‐free promoter vector (InvivoGen). The putative enhancers were amplified from DNA samples using oligonucleotides containing the required restriction enzyme sequences for cloning (Supplementary Table 6, available on the *Arthritis & Rheumatology* website at https://onlinelibrary.wiley.com/doi/10.1002/art.42427). The PCR products were cloned into the vector as previously described (21, 22). Plasmids were methylated or mock‐methylated in vitro using *M.SssI* (New England BioLabs). Cells from the human chondrocyte cell line Tc28a2 (30) were seeded at 5,000 cells/well in a 96‐well plate and transfected with 100 ng pCpG‐free promoter constructs and 10 ng pGL3‐promoter control vector (Promega) using Lipofectamine 2000 (Invitrogen). Cells were lysed after 24 hours, and luminescence was read using the Dual‐Luciferase Reporter Assay System (Promega) and analyzed as previously described (21).

### Epigenetic modulation

Guide RNA 1 (gRNA1) and gRNA2, targeting cg15204595 and cg21606956, respectively, were designed using the CRISPR/Cas9 guide RNA design tool (IDT). The gRNA sequences (Supplementary Table 6, available at https://onlinelibrary.wiley.com/doi/10.1002/art.42427) were synthesized as single‐stranded cDNA oligonucleotides (IDT) with overhangs to facilitate cloning. For methylation, oligonucleotides were annealed and ligated into pdCas9‐DNMT3a‐EGFP plasmid (31) (Addgene, plasmid no. 71666) and the catalytically inactivated control plasmid pdCas9‐DNMT3a‐EGFP (ANV) (31) (Addgene, plasmid no. 71685) as previously described (21, 22). For demethylation, the pdCas9‐DNMT3a‐EGFP plasmids containing the gRNAs were digested with *PvuI* and *XbaI* (New England Biolabs), and scaffold regions were subcloned into pSpdCas9‐huTET1CD‐T2A‐mCherry plasmid (Addgene, plasmid no. 129027) and the catalytically inactivated control plasmid pSpdCas9‐hudTET1CD‐T2A‐mCherry (Addgene, plasmid no. 129028), as previously described (31). Each construct (5 μg) was nucleofected into 1 × 10^6^ Tc28a2 cells using the 4D Nucleofector kit (Lonza), with transfection confirmed after 24 hours by green fluorescent protein (for DNMT3a plasmids) or mCherry (for TET1 plasmids) visualization (Zeiss AxioVision).

Cells were harvested 72 hours after transfection. Nucleic acids were extracted using a DNA/RNA Purification Kit (Norgen Biotek). DNAm levels at cg15204595 and cg21606956 were measured using pyrosequencing. RNA (500 ng) was reverse transcribed using SuperScript IV Reverse Transcriptase (Invitrogen), and gene expression was measured by reverse transcription–quantitative PCR using Quant Studio 3 (Applied Biosystems). The expression of *COLGALT2* and *TSEN15*, normalized to that of housekeeping genes *18S, GAPDH*, and *HPRT1*, was calculated using the 2^−ΔCt^ method (32). TaqMan assays were purchased from IDT (Supplementary Table 7, available on the *Arthritis & Rheumatology* website at https://onlinelibrary.wiley.com/doi/10.1002/art.42427).

### Statistical analysis

Wilcoxon's matched pairs signed rank test was used to calculate *P* values in AEI analysis. For graphical representations of DNAm data, methylation status was plotted in the form of β‐values, ranging from 0 (no methylation) to 1 (100% methylation). For statistical analysis of methylation data, β‐values were converted to M‐values (33). In mQTL analysis, linear regression was used to assess the relationship between CpG methylation and genotype (0, 1, or 2 copies of the minor allele) at rs1046934. For mQTL discovery, these calculations were performed using the Matrix eQTL package (34) in R, with age, sex, and joint site (hip or knee) used as covariates. Associations between AEI and DNAm and between expression of TFs and *COLGALT2* were determined using linear regression. Mann‐Whitney U test was used to calculate *P* values when comparing methylation levels irrespective of genotype. For Lucia reporter gene assays, *P* values were calculated by paired and unpaired *t*‐tests. Paired *t*‐tests were used to calculate *P* values for changes in gene expression following epigenetic modulation. Unless stated otherwise, statistical tests were performed in GraphPad Prism.

## RESULTS

### Missense variants not predicted to affect the TSEN15 protein

**Figure 1:**
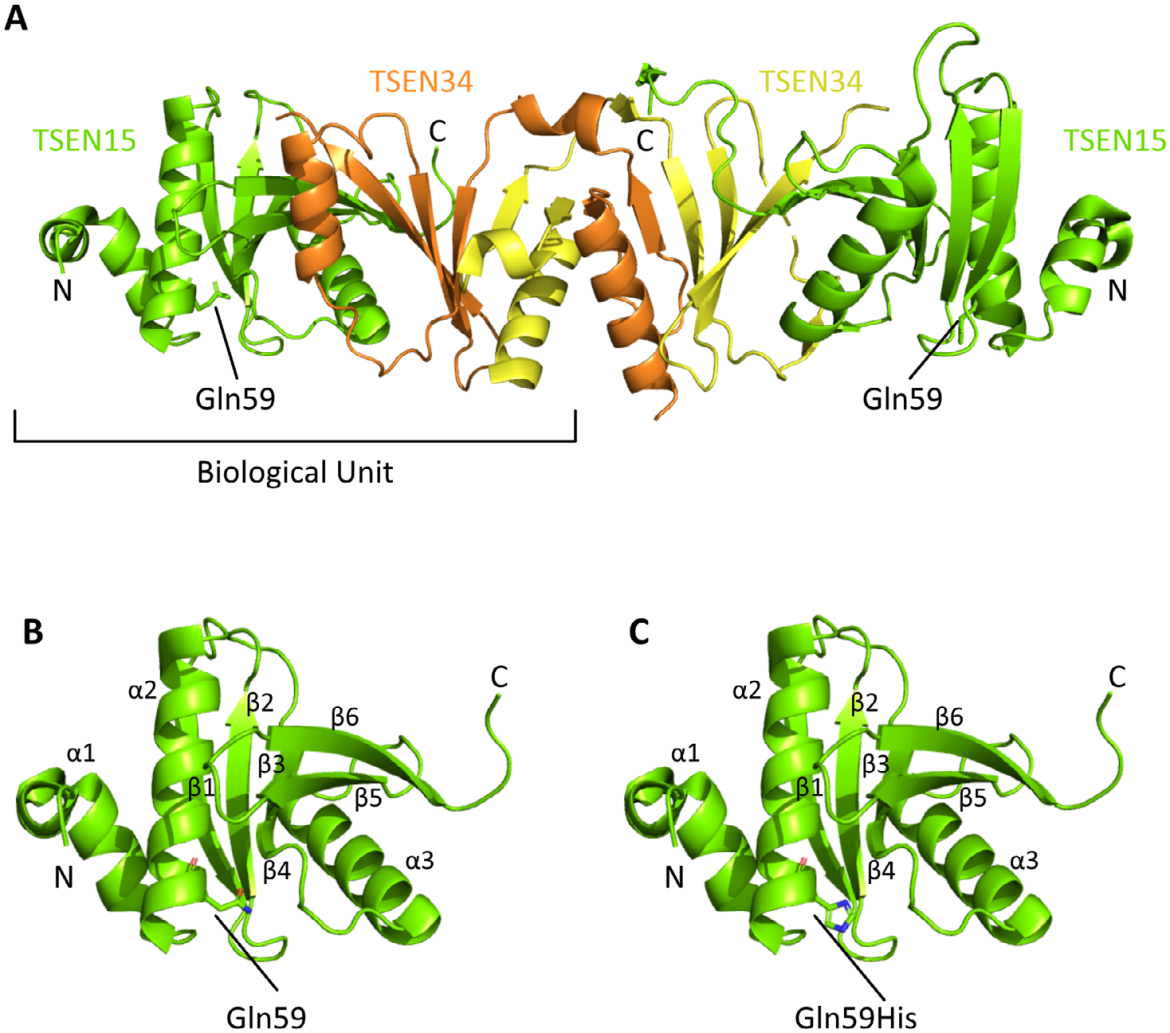
TSEN15 protein structure. **A,** Crystal structure of the TSEN15 (green)–TSEN34 (yellow and orange) heterodimer (Protein Data Bank, identification no. 6Z9U). The position of the Gln^59^ residue is highlighted, and the amino acid side chain is displayed. **B,** Monomeric crystal structure of TSEN15 (Protein Data Bank, identification no. 2GW6), showing numbering of the α‐α‐β‐β‐β‐β‐α‐β‐β fold (α_1–3_ and β_1–6_). Gln^59^ is labeled, and the side chain is displayed. **C,** TSEN15 structure, shown as in **B**, following in silico mutagenesis to predict the conformation of Gln^59^‐His (labeled). In **B** and **C**, red shows oxygen atoms and blue shows nitrogen atoms. The PyMOL Molecular Graphics System was used to view structures and to perform mutagenesis.

The rs1046934 locus encompasses transcript SNPs that introduce amino acid (missense) substitutions into TSEN15: rs1046934 itself (A>C, pGln^59^–His) and rs2274432 (G>A, pGly^19^‐Asp). These SNPs are in perfect LD (r^2^ = 1). Gln^59^ falls within the α_2_‐helix of TSEN15 (Figures 1A and 1B). In silico mutagenesis of the residue predicts an outward‐facing position of the histidine side chain, away from the coiled‐coil interactions between the α_1_‐helix and α_2_‐helix (Figure 1C). This indicates that the variant is unlikely to affect the structure or stability of TSEN15. We could not undertake in silico mutagenesis of Gly^19^ since the Gly^19^–Asp variant resides within the structurally unresolved N‐terminal region of TSEN15. However, gnomAD, PolyPhen, and Mutation Taster all predicted this variant, as well as Gln^59^‐His, as benign. We conclude that the risk of OA residing at the rs1046934 locus is not driven by changes to TSEN15 protein function.

### Association between the genotype at rs1046934 and expression of *COLGALT2* and *TSEN15* in human arthroplasty cartilage

The rs1046934 OA association signal and the observation that the genotype at the SNP is associated with expression of *COLGALT2* and *TSEN15* were reported in a range of tissues in the Genotype‐Tissue Expression (GTEx) portal, forming eQTLs (4). None of the tissues comprising the GTEx portal originate from articulating joints. Therefore, we undertook an AEI analysis in cartilage samples from OA patients to assess whether the rs1046934 genotype was associated with expression of either *COLGALT2* or *TSEN15* in this disease relevant tissue.

**Figure 2:**
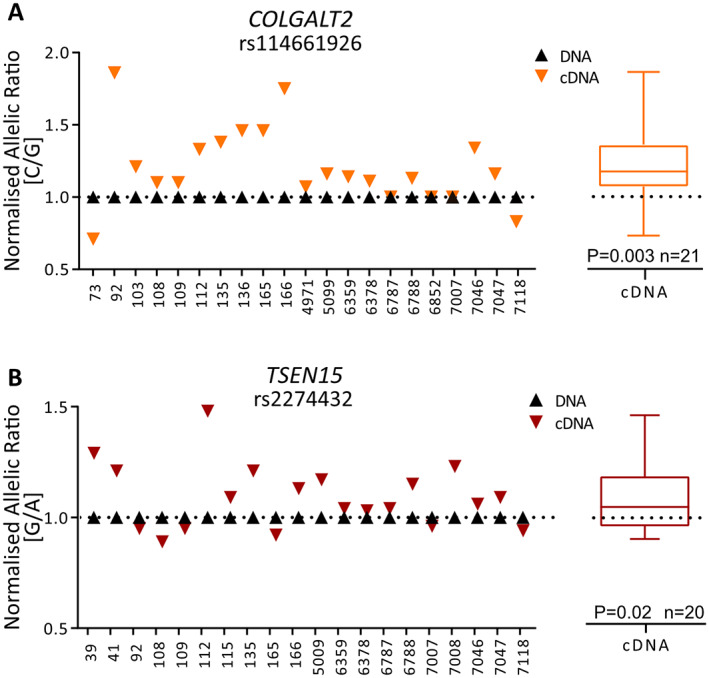
Allelic expression imbalance analysis of *COLGALT2* and *TSEN15* in human arthroplasty cartilage samples. **A**, Allelic ratios for *COLGALT2* transcript single‐nucleotide polymorphism (SNP) rs114661926 (C/G; C = osteoarthritis [OA] risk allele). **B**, Allelic ratios for *TSEN15* transcript SNP rs2274432 (G/A; G = OA risk allele). Numbers on x‐axes indicate patient sample identification numbers. Symbols in graphs represent the mean of 3 technical replicates. Mean complementary DNA (cDNA) values measured across all samples are shown as box plots. Each box represents the interquartile range. Lines inside the box represent the median. Whiskers represent the minimum and maximum values. Dashed lines represent the allele ratios in DNA. *P* values were calculated using Wilcoxon's matched pairs signed rank test. Color figure can be viewed in the online issue, which is available at http://onlinelibrary.wiley.com/doi/10.1002/art.42427/abstract.

Both genes demonstrated AEI (Figure 2), with OA risk allele C of *COLGALT2* transcript SNP rs114661926 showing an average 1.21‐fold increase in *COLGALT2* expression (*P* = 0.003) and OA risk allele G of *TSEN15* transcript SNP rs2274432 showing an average 1.09‐fold increase in *TSEN15* expression (*P* = 0.02).

### Identification of rs1046934 mQTLs operating within putative enhancers in human arthroplasty cartilage

**Figure 3:**
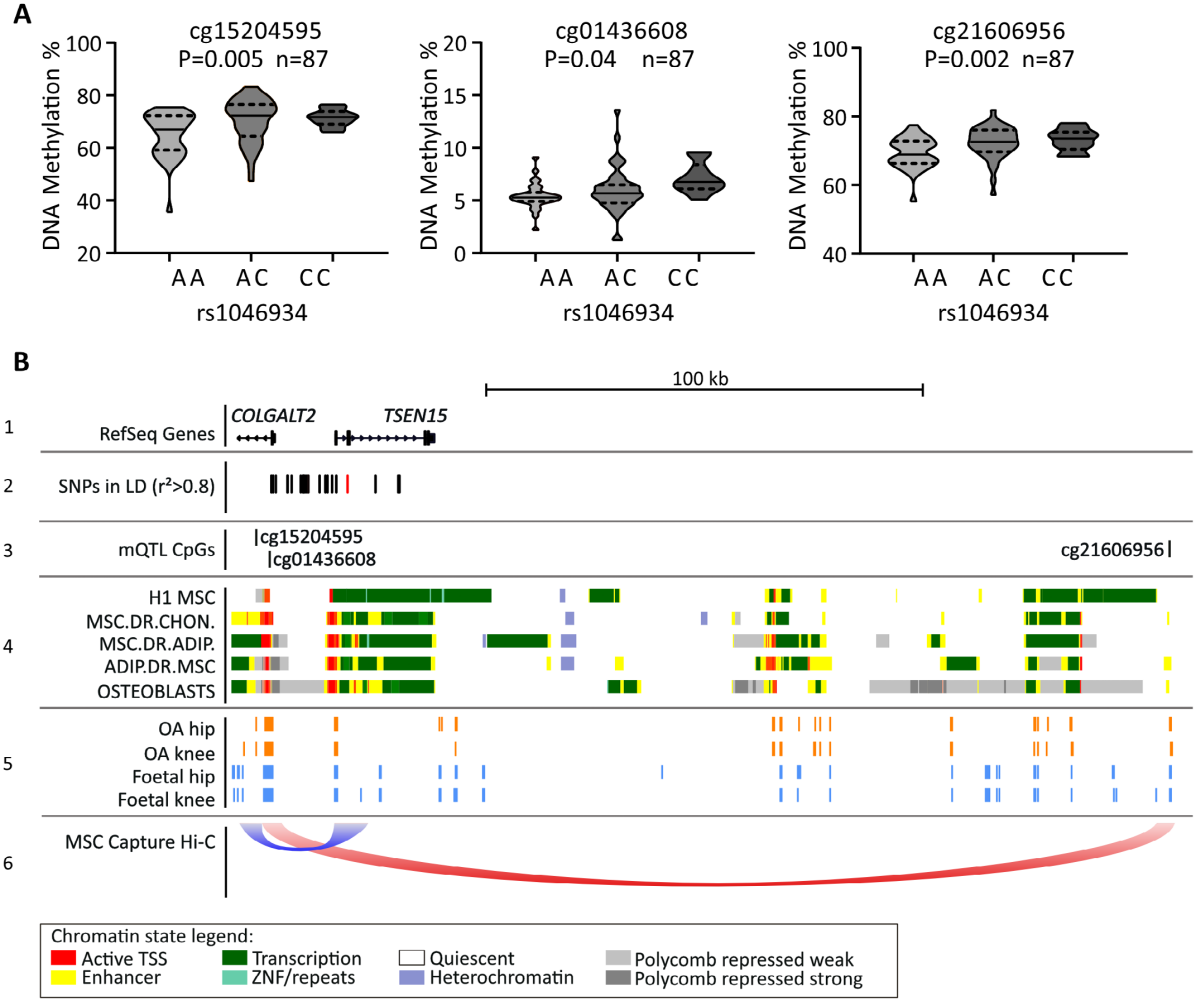
Methylation quantitative trait locus (mQTL) discovery and in silico analysis. **A,** DNA methylation (DNAm) values at cg15204595, cg01436608, and cg21606956 stratified by rs1046934 genotype, using hip or knee arthroplasty cartilage samples from osteoarthritis (OA) patients (n = 87), are shown as violin plots. Solid lines and dashed lines inside the plots represent median and interquartile range, respectively. *P* values were calculated by linear regression. **B,** Schematic overview of the rs1046934 locus. Panel 1, Relative genomic position of the 5' end of *COLGALT2* and all of *TSEN15*, visualized using the University of California Santa Cruz Genome Browser (hg19). Panel 2, Red line indicates position of rs1046934, and black lines indicate single‐nucleotide polymorphisms (SNPs) in high linkage disequilibrium (LD) (pairwise r^2^ > 0.8). SNPs comprise a 30‐kb block. Panel 3, Black lines indicate positions of cg15204595, cg01436608, and cg21606956. Panel 4, Chromatin state data, as determined from the Roadmap project, for primary human mesenchymal stem cells (H1 MSC), MSC‐derived chondrocytes (MSC.DR.CHON) and adipocytes (MSC.DR.ADIP), adipose‐derived MSCs (ADIP.DR.MSC), and human osteoblasts. Colors correspond to different chromatin states, as indicated in legend at bottom. Panel 5, Open regions marked by orange blocks indicate ATAC‐sequencing peaks generated from OA hip and knee chondrocytes; open regions marked by blue blocks indicate ATC‐sequencing peaks generated from fetal hip and knee chondrocytes. Panel 6, Loops indicate capture Hi‐C chromatin interactions from the 3D Genome Browser in human MSCs, with the flat ends of loops spanning the width of the interacting regions. TSS = transcription start site. Color figure can be viewed in the online issue, which is available at http://onlinelibrary.wiley.com/doi/10.1002/art.42427/abstract.

We next analyzed an arthroplasty cartilage epigenome‐wide DNAm data set derived from the cartilage of 87 OA patients (28) to assess whether the rs1046934 genotype was associated with proximal DNAm levels. We analyzed 58 CpGs in a 400‐kb interval surrounding rs1046934 (Supplementary Table 8, available on the *Arthritis & Rheumatology* website at https://onlinelibrary.wiley.com/doi/10.1002/art.42427) and identified 3 CpGs with methylation status that was nominally (*P* < 0.05) associated with genotype, forming mQTLs: cg15204595 (*P* = 0.005), cg01436608 (*P* = 0.04), and cg21606956 (*P* = 0.002). At all 3 CpGs, the OA risk allele A of rs1046934 was associated with reduced methylation (Figure 3A).

We observed that rs1046934 and the 20 SNPs in high pairwise LD (r^2^ > 0.8) form a 30‐kb block that encompasses the 5′‐untranslated region and promoter of *COLGALT2*, the promoter and part of the gene body of *TSEN15*, and the intergenic region between the 2 genes (Figure 3B, panels 1 and 2). Two of the mQTL CpGs, cg15204595 and cg01436608, are 2.35‐kb apart and located within intron 1 of *COLGALT2* (Figure 3B, panels 1 and 3). Both are close to the LD block, with cg01436608 being 595 bp from rs74767794, the most upstream variant in the block. Both cg15204595 and cg01436608 reside within a region that is marked as an enhancer and a transcriptionally active site in a range of relevant human cell types (primary MSCs, MSC‐derived chondrocytes and adipocytes, adipose‐derived MSCs, and primary osteoblasts) (Figure 3B, panel 4) and marked as an open chromatin region in OA and fetal chondrocytes (Figure 3B, panel 5). Conversely, cg21606956 is distal to the LD block and over 200 kb from cg15204595 and cg01436608 (Figure 3B, panels 1–3), falling within an intergenic enhancer (Figure 3B, panels 3 and 4) that is marked as an open chromatin region in OA and fetal chondrocytes (Figure 3B, panel 5). MSC capture Hi‐C data showed physical interactions between a broad region encompassing rs1046934 and the enhancer containing cg15204595 (Figure 3B, panel 6). Additional interactions were observed between the *COLGALT2* promoter and the enhancer containing cg21606956 (Figure 3B, panel 6).

### Replication of mQTLs using cartilage DNA from an independent human arthroplasty cohort

**Figure 4:**
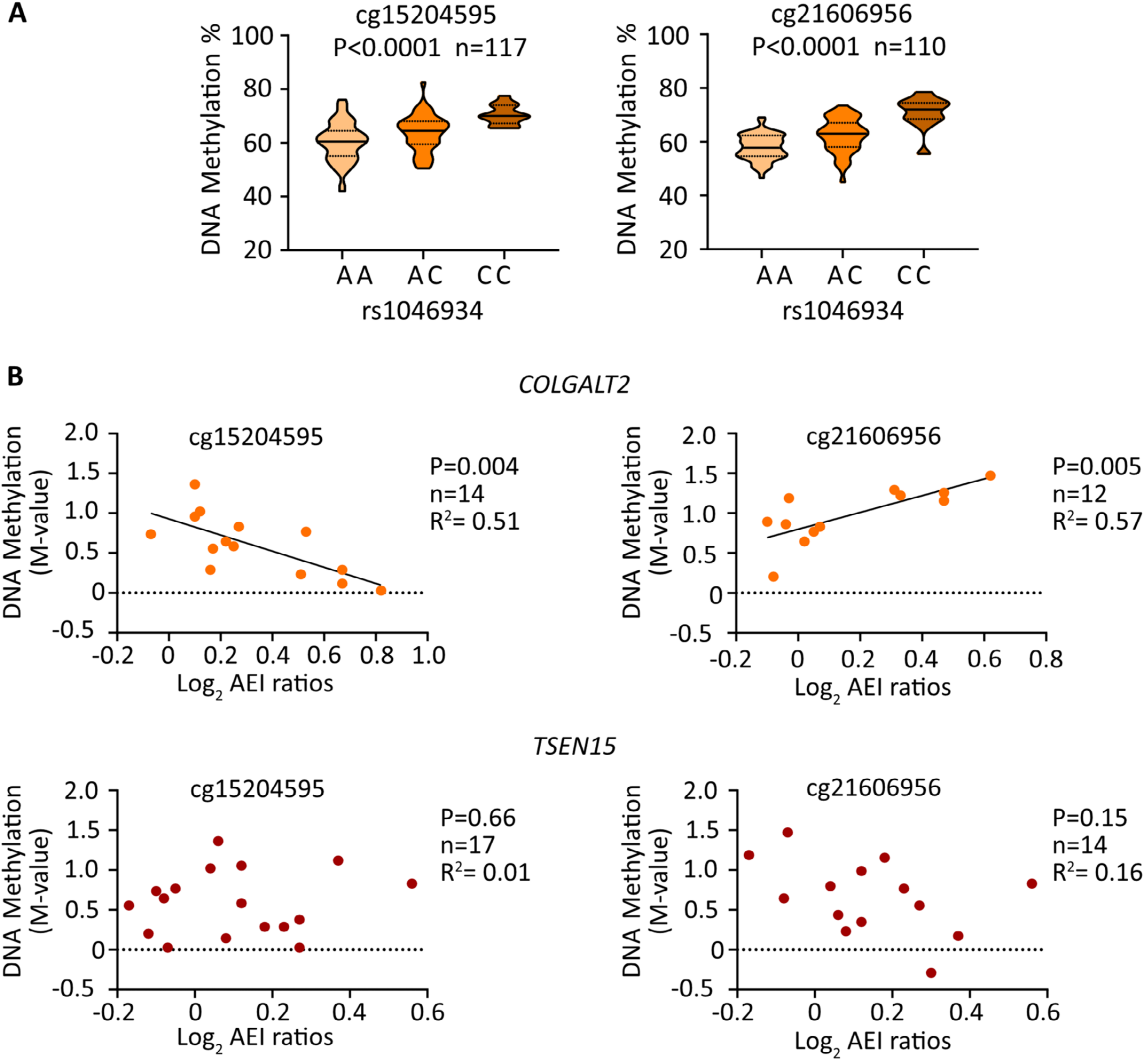
Replication of mQTLs and discovery of methylation–expression QTLs (meQTLs) in arthroplasty cartilage. **A,** DNAm values at cg15204595 and cg21606956 stratified by genotype at rs1046934, using DNA derived from cartilage from hip and knee OA patients and femoral neck fracture patients, are shown as violin plots. Solid lines and dashed lines inside the plots represent the median and interquartile range, respectively. *P* values were calculated by linear regression. **B,** Graphs show allelic expression imbalance (AEI) allelic ratios (log_2_) for *COLGALT2* (rs114661926) and *TSEN15* (rs2274432) plotted against matched DNAm levels (M‐values) at cg15204595 and cg21606956. Symbols represent data from 1 individual. *P* values were calculated by linear regression. See Figure 3 for other definitions. Color figure can be viewed in the online issue, which is available at http://onlinelibrary.wiley.com/doi/10.1002/art.42427/abstract.

Because none of the CpGs from the analysis of the 87 patients would have significant *P* values following multiple correction testing, we set out to replicate the 3 mQTLs in an independent cohort of arthroplasty cartilage DNAs. We were able to design pyrosequencing assays for cg15204595 and cg21606956 but not for cg01436608, due to a long run of thymine bases following bisulfite conversion and subsequent PCR amplification. The cg15204595 and cg21606956 mQTLs replicated (*P* < 0.0001 for each) and confirmed the association between the OA risk allele A of rs1046934 and reduced methylation (Figure 4A).

The cartilage DNAs used for replication were derived from OA (hip and knee) patients and femoral neck fracture patients. After stratification by disease state (OA or femoral neck fracture; Supplementary Figure 1A, available on the *Arthritis & Rheumatology* website at https://onlinelibrary.wiley.com/doi/10.1002/art.42427), mQTLs were detectable in both patient groups, indicating that differential methylation is not a consequence of OA disease state. When the data were stratified by disease state irrespective of rs1046934 genotype (Supplementary Figure 1B), methylation at cg15204595 was significantly higher in the femoral neck fracture group than in the OA group (*P* = 0.003). Patients with femoral neck fracture were on average older at surgery (77.35 years of age) than patients with OA at surgery (65.32 years of age for knee OA and 66.51 years of age for hip OA) (Supplementary Table 2, available at https://onlinelibrary.wiley.com/doi/10.1002/art.42427). We found no significant association between age and DNAm status (*P* > 0.05; Supplementary Figure 2, available at https://onlinelibrary.wiley.com/doi/10.1002/art.42427).

### Association between CpG methylation and *COLGALT2* expression

We subsequently assessed whether there were associations between DNAm and gene expression in samples with matched data (Figure 4B). For *COLGALT2*, significant associations were observed at cg15204595 (r^2^ = 0.51, *P* = 0.004) and cg21606956 (r^2^ = 0.57, *P* = 0.005), marking methylation–expression QTLs (meQTLs). No significant associations were shown between either of the CpGs and *TSEN15*.

### Identification of OA genetic risk mechanisms at the rs1046934 locus in human fetal development

As noted earlier, we recently reported that, for several OA SNPs, the associations between gene expression and CpG methylation observed in human arthroplasty cartilage also occur in fetal cartilage (24). Therefore, we determined whether the rs1046934 AEI, mQTL, and meQTL effects were also detectable in fetal cartilage.

**Figure 5:**
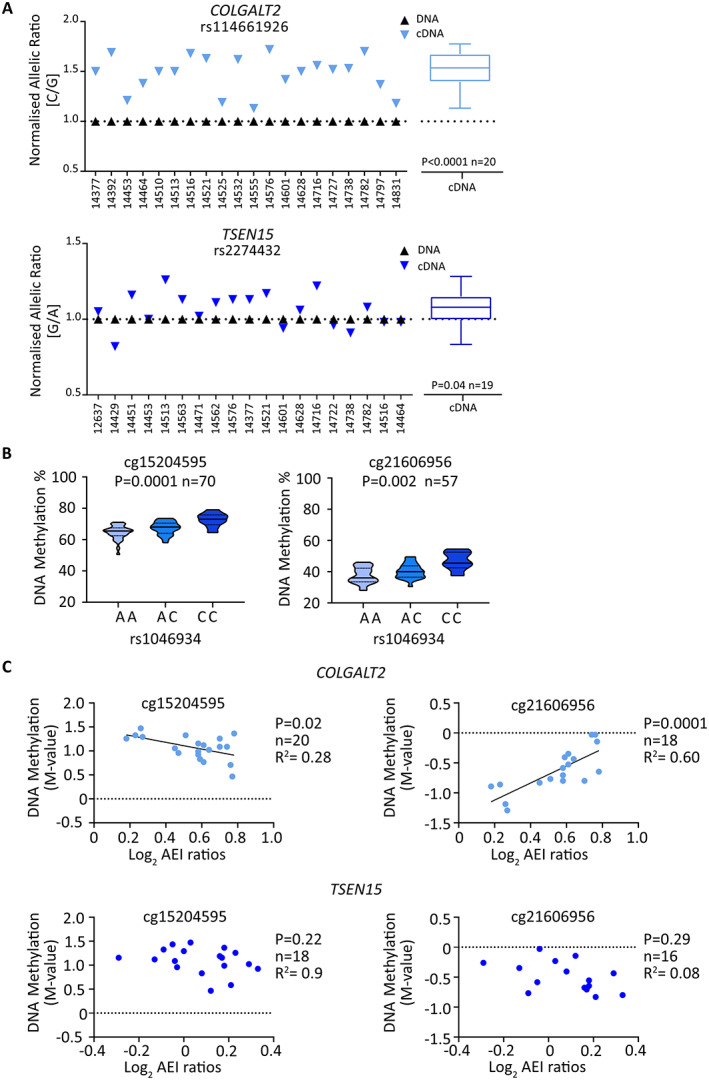
AEI, mQTL, and meQTL analyses in fetal cartilage. **A,** Allelic ratios for *COLGALT2* transcript SNP rs114661926 (C/G; C = OA risk allele) and for *TSEN15* transcript SNP rs2274432 (G/A; G = OA risk allele). Numbers on x‐axes indicate patient sample identification numbers. Symbols in graphs represent the mean of 3 technical replicates. Mean complementary DNA (cDNA) values measured across all samples are shown as box plots. Each box represents the interquartile range. Lines inside the box represent the median. Whiskers represent the minimum and maximum values. Dashed lines represent the allele ratios in DNA. *P* values were calculated using Wilcoxon's matched pairs signed rank test. **B,** DNAm values at cg15204595 and cg21606956 stratified by genotype at rs1046934, using DNA derived from fetal cartilage, are shown as violin plots. Solid lines and dashed lines inside the plots represent the median and interquartile range, respectively. *P* values were calculated by linear regression. **C,** Graphs show AEI allelic ratios (log_2_) for *COLGALT2* (rs114661926) and *TSEN15* (rs2274432) plotted against matched DNAm levels (M‐values) at cg15204595 and cg21606956. Symbols represent data from 1 individual. *P* values were calculated by linear regression. See Figure 3 for other definitions. Color figure can be viewed in the online issue, which is available at http://onlinelibrary.wiley.com/doi/10.1002/art.42427/abstract.

AEI was detected for both genes (Figure 5A) in fetal cartilage, in the same direction as that observed in human arthroplasty cartilage (Figure 2), with OA risk allele C at rs114661926 showing an average 1.35‐fold increase in *COLGALT2* expression (*P* < 0.0001) and OA risk allele G at rs2274432 showing an average 1.03‐fold increase in *TSEN15* expression (*P* = 0.04).

In fetal DNA, cg15204595 and cg21606956 displayed mQTL effects (Figure 5B) in the same direction as observed in human arthroplasty DNA (Figure 4A), with OA risk allele A of rs1046934 showing an association with reduced methylation. Mean DNAm levels at cg15204595 were higher in fetal cartilage (66.7%) than in arthroplasty cartilage (62.8%) (*P* = 0.0002), with the opposite observed at cg21606956, with mean values of 40.0% in fetal cartilage and 61.1% in arthroplasty cartilage (*P* < 0.0001) (Supplementary Figure 3, available on the *Arthritis & Rheumatology* website at https://onlinelibrary.wiley.com/doi/10.1002/art.42427). In fetal cartilage, meQTLs were observed for *COLGALT2* but not for *TSEN15* (Figure 5C), consistent with our observations in arthroplasty cartilage (Figure 4B).

In both arthroplasty and fetal cartilage, the slopes of the *COLGALT2* meQTLs at cg15204595 and cg21606956 were in opposite directions. At cg15204595, high M‐values were associated with low AEI ratios, whereas, for cg21606956, high M‐values were associated with high AEI ratios (Figure 4B, Figure 5C). A proposed model describing this is presented in the Supplementary Text and in Supplementary Figure 4, available on the *Arthritis & Rheumatology* website at https://onlinelibrary.wiley.com/doi/10.1002/art.42427.

### Presence of cg15204595 and cg21606956 in enhancers and effects of their demethylation on *COLGALT2* expression

**Figure 6:**
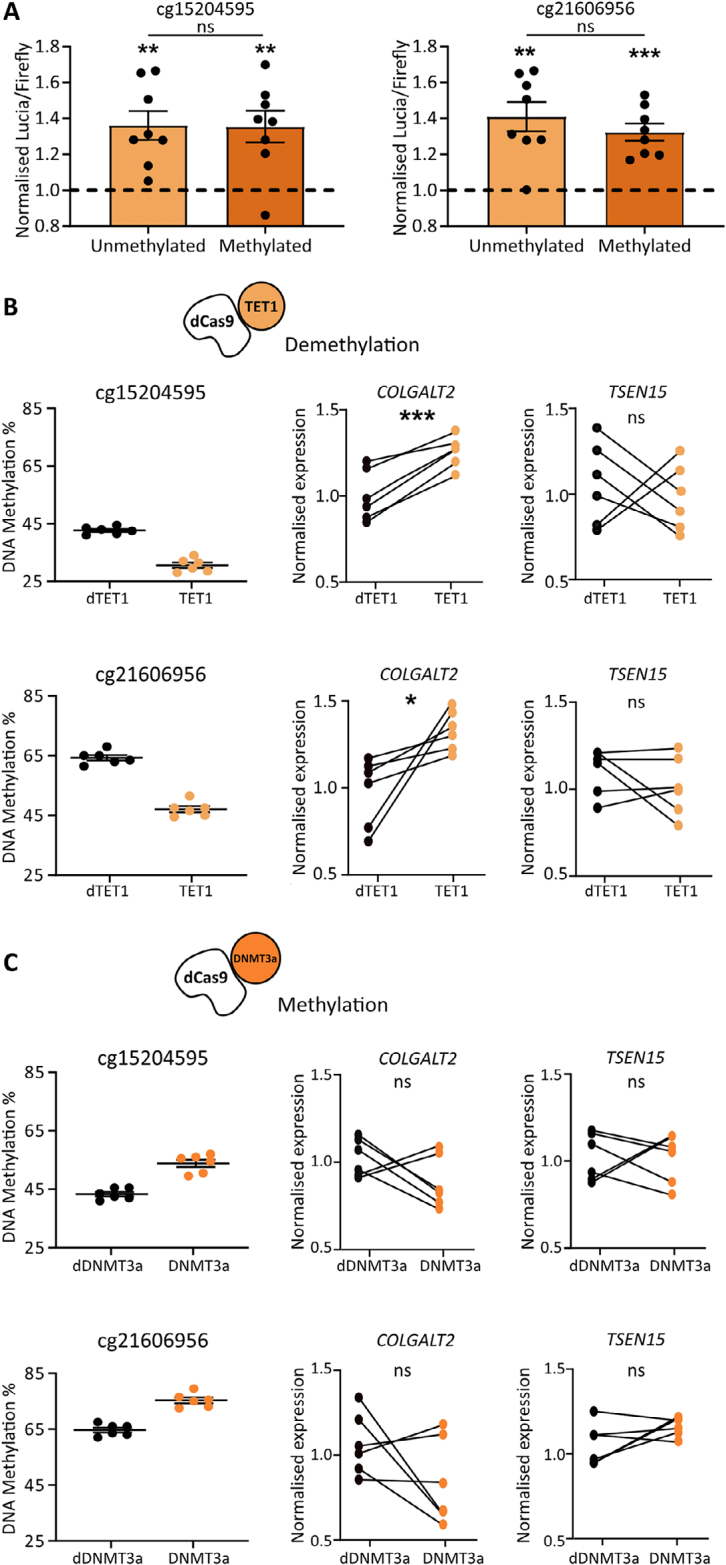
cg15204595 and cg21606956 reside in enhancers and increase *COLGALT2* expression when demethylated. **A,** Lucia luminescence in Tc28a2 chondrocytes following transfection with constructs containing cg15204595 (left) or cg21606956 (right) in unmethylated or methylated states. Symbols represent biologic replicates (n = 8). Dashed lines represent cells with empty control vectors. **B,** Left, DNAm levels at cg15204595 (top) and cg21606956 (bottom) in Tc28a2 chondrocytes following transfection of guide RNAs (gRNAs) with dead Cas9 (dCas9) coupled with dead TET1 (dTET1) in controls (black symbols) or with active TET1 (orange symbols) (n = 6 biologic replicates/treatment). Right, Effect of demethylation on gene expression. Values were normalized to mean control values. **C,** Left, DNAm levels at cg15204595 (top) and cg21606956 (bottom) in Tc28a2 chondrocytes following transfection of gRNAs with dCas9 coupled with dead DNMT3a (dDNMT3a) in controls (black symbols) or with active DNMT3a (orange symbols) (n = 6 biologic replicates/treatment). Right, Effect of methylation on gene expression. Values were normalized to mean control values. **A**–**C**, Bars show mean ± SEM. *P* values were calculated using paired *t*‐test for empty control versus insert and unpaired *t*‐test for unmethylated versus methylated insert (**A**) or were calculated using paired *t*‐test (**B** and **C**). * = *P* < 0.05; ** = *P* < 0.01; *** = *P* < 0.001; ns = not significant (*P* > 0.05). See Figure 3 for other definitions. Color figure can be viewed in the online issue, which is available at http://onlinelibrary.wiley.com/doi/10.1002/art.42427/abstract.

Using the chondrocyte cell line Tc28a2, we cloned the regions surrounding cg15204595 and cg21606956 into the CpG‐free Lucia reporter gene vector and tested for enhancer activity in methylated and unmethylated states. No other CpGs were captured within the cloned regions. For cg15204595, the unmethylated and methylated constructs showed increased Lucia readings compared with that shown in reporter gene assays with the empty control vectors, with an average increase in activity of 1.36‐fold (*P* < 0.01) and 1.35‐fold (*P* < 0.01) respectively (Figure 6A, left). The region encompassing cg21606956 also acted as an enhancer, with an average 1.41‐fold (*P* < 0.01) and 1.32‐fold (*P* < 0.001) increase in Lucia activity in the unmethylated and methylated constructs, respectively (Figure 6A, right). In vitro methylation status had no significant effect on the function of the enhancers.

Targeted demethylation and methylation of cg15204595 and cg21606956 in Tc28a2 cells was performed to investigate the effects of DNAm on *COLGALT2* and *TSEN15* expression using catalytically dead Cas9 (dCas9) protein coupled with catalytically active TET1 (to demethylate) or DNMT3a (to methylate). Control cells were transfected with the same gRNAs coupled with dCas9 and dead TET1 (dTET1) or dead DNMT3a (dDNMT3a). Mean reductions in methylation at cg15204595 of 12.8% and cg21606956 of 17.3% were achieved using TET1 (Figure 6B, left). This resulted in 1.3‐fold (*P* = 0.0009) and 1.2‐fold (*P* = 0.01) increases in *COLGALT2* expression but no significant change in *TSEN15* expression (Figure 6B, right). Mean increases in methylation at cg15204595 of 10.5% and cg21606956 of 10.7% were achieved using DNMT3a (Figure 6C, left). Increases in methylation did not significantly alter expression of either gene (Figure 6C, right).

### Investigation of the OA risk SNPs affecting TF binding at the locus

Noncoding SNPs can mediate their effects on expression of a target gene through alteration of the consensus sequence of DNA binding proteins, including TFs (5, 6, 7, 8). This variance can lead to differential protein binding that directly or indirectly (i.e., with a DNAm intermediary) alters transcription (5, 6, 7, 8). One of the major hurdles of post‐GWAS functional studies is identifying the causal variants within the risk haplotype, marked by the association signal. These SNPs can exert their functional effects in concert through shared or distinct mechanisms (5, 6, 7, 8).

To investigate which of the 21 SNPs in the rs1046934 LD block may alter TF binding, we used SNP2TFBS (Supplementary Table 1, available at https://onlinelibrary.wiley.com/doi/10.1002/art.42427). Six SNPs were identified that were predicted to impact the binding of 8 TFs, 4 of which are expressed in human arthroplasty cartilage (transcripts per million >10) (Supplementary Table 9, available at https://onlinelibrary.wiley.com/doi/10.1002/art.42427). The expression levels of these genes (*STAT1, STAT2, SP2*, and *ZNF263*) were regressed against *COLGALT2* expression (Supplementary Figure 5, available at https://onlinelibrary.wiley.com/doi/10.1002/art.42427). A significant association was identified between *COLGALT2* and *SP2* (*P* = 0.001). Interestingly, 1 of the 2 SNPs predicted to disrupt SP2 binding (rs74767794) is located within the *COLGALT2* promoter. This potentially points toward an additional, direct genetic impact on *COLGALT2* conferred by the risk haplotype.

Our targeted epigenetic modulation demonstrated that demethylation of cg15204595 and cg21606956 has direct effects on the function of their respective enhancer regions (Figure 6B). Methylation at CpGs has the potential to alter the binding efficiency of TFs to DNA, modulating enhancer activity (35, 36). We hypothesized that these CpGs also fall within protein binding motifs and can influence TF recruitment to the enhancers. To assess this, we searched the JASPAR database (Supplementary Table 1, available at https://onlinelibrary.wiley.com/doi/10.1002/art.42427) and identified multiple TFs predicted to bind at or near the CpGs (Supplementary Figures 6A and B, available at https://onlinelibrary.wiley.com/doi/10.1002/art.42427), many of which are expressed in human cartilage (Supplementary Figure 6C).

## DISCUSSION

We used a range of techniques to study a novel OA association locus marked by rs1046934. This signal maps close to *COLGALT2*, a gene that we had previously highlighted as a target of a completely independent OA risk locus, marked by rs11583641 (22). We discovered that the rs1046934 locus, like the rs11583641 locus, mediates its effect by modulating the expression of *COLGALT2* via methylation changes to CpGs located in enhancers. The associated SNPs, the CpGs, and the enhancers are entirely distinct between the 2 loci, but their ultimate effect on *COLGALT2* is the same.

Our analysis of human arthroplasty cartilage showed that the OA risk allele A of rs1046934 was associated with increased *COLGALT2* expression and decreased methylation of CpGs cg15204595, cg01436608, and cg21606956, with the methylation effects observed at cg15204595 and cg21606956 replicated in an independent cohort. Importantly, we identified significant associations between methylation and *COLGALT2* expression. Epigenetic modulation demonstrated this to be a direct causal link, with demethylation increasing expression. Furthermore, reporter gene assays confirmed that the genomic regions harboring cg15204595 and cg21606956 are enhancers in chondrocytes. In silico data revealed that the CpGs reside in or close to TF binding sites and in open chromatin regions in chondrocytes, further supporting their functional role. MSC capture Hi‐C data highlighted the physical interactions encompassing the associated SNPs, the cg15204595 and cg21606956 enhancers, and the *COLGALT2* promoter. Therefore, we conclude that these enhancers interact with *COLGALT2* to regulate its expression, with genotype at the association signal modulating the methylation status and consequently the function of the enhancers.

Although OA is a disease of older people, OA susceptibility has been reported to have developmental origins, with many OA SNPs associating with joint shape phenotypes (38, 39, 40, 41, 42, 43). This implies that a proportion of OA genetic risk is functionally active during skeletogenesis and early postnatal life and manifests with aging (44, 45, 46). We previously investigated this by assessing AEI and mQTLs at OA risk loci in fetal cartilage samples (24). For a proportion of the studied loci, the AEI and mQTLs observed in arthroplasty cartilage were also observed in fetal cartilage (24). This included the rs11583641 *COLGALT2* locus (24), which prompted us to investigate fetal cartilage at the rs1046934 locus. The rs1046934 AEI, mQTL, and meQTL effects detected in human arthroplasty cartilage were also detected in fetal cartilage, implying that this locus is one in which the molecular effects on a target gene are activated during development. Our cohort of arthroplasty cartilage samples included cartilage from patients with femoral neck fracture. Although femoral neck fracture patients lack OA cartilage lesions in their hip joints, we detected mQTLs at cg15204595 and cg21606956 in their DNA. Combined, our fetal and femoral neck fracture data imply that the molecular effects of the rs1046934 signal on *COLGALT2* are not dependent on age or OA disease status yet contribute to this highly polygenic disease across the life course.

In our dCas9 experiment, demethylation of cg15204595 and cg21606956 had significant effects on *COLGALT2* expression. Demethylating cg15204595 and cg21606956 in vitro mimics the effect of the risk‐conferring allele A of rs1046934 in cartilage, which is associated with reduced methylation of the CpGs and with increased *COLGALT2* expression. We propose that the enhancers harboring cg15204595 and cg21606956 are particularly sensitive to decreased methylation, accounting for the changes in *COLGALT2* expression, which were only measured when DNAm levels were reduced and not increased.

Throughout our study, we investigated *TSEN15* alongside *COLGALT2*, as both genes were highlighted in the discovery GWAS as potential targets of the association signal, primarily due to rs1046934 eQTLs shown at each gene in the GTEx portal (4). We observed AEI at *TSEN15*, albeit the fold differences in expression between risk and nonrisk alleles were not as large as those for *COLGALT2*. However, we did not observe meQTLs for *TSEN15*, and the epigenetic modulation of cg15204595 and cg21606956 did not significantly alter *TSEN15* expression. Furthermore, our in silico analyses of the *TSEN15* missense variants did not indicate that the changes affected protein structure or function. Despite these observations, we cannot definitively exclude *TSEN15* as an additional target of the association signal.

Clinical exploitation of OA genetic discoveries will require an understanding of the molecular mechanism by which risk‐conferring alleles impact their target genes (1, 46, 47). In this study, we undertook a detailed analysis of the OA locus marked by rs1046934, highlighting its effect on the expression of *COLGALT2* via 2 distal enhancers that are epigenetically regulated. For the first time to our knowledge, our data provide compelling evidence of a target gene being impacted in a nearly identical manner by 2 genetically independent OA association signals and disease‐relevant gene enhancers. This increases confidence in *COLGALT2* and its encoded enzyme as targets of OA risk and therefore prioritizes them for future translational investigation.

## AUTHOR CONTRIBUTIONS

All authors were involved in drafting the article or revising it critically for important intellectual content, and all authors approved the final version to be published. Prof. Loughlin had full access to all of the data in the study and takes responsibility for the integrity of the data and the accuracy of the data analysis.

### Study conception and design

Wilkinson, Rice, Loughlin.

### Acquisition of data

Kehayova, Rice.

### Analysis and interpretation of data

Kehayova, Wilkinson, Rice, Loughlin.

## Supporting information


Disclosure Form

**Appendix S1:** Supplementary Information
