# Supplementary material for: Mediation of the Same Epigenetic and Transcriptional Effect by Independent Osteoarthritis Risk–Conferring Alleles on a Shared Target Gene, COLGALT2
Source: Arthritis Rheumatol. 2023 Apr 11;75(6):910–22. doi: 10.1002/art.42427 (PMC10952352; doi:10.1002/art.42427)
Supplement: Supplementary file 2 — Appendix S1: Supplementary Information [file ART-75-910-s002.pdf]

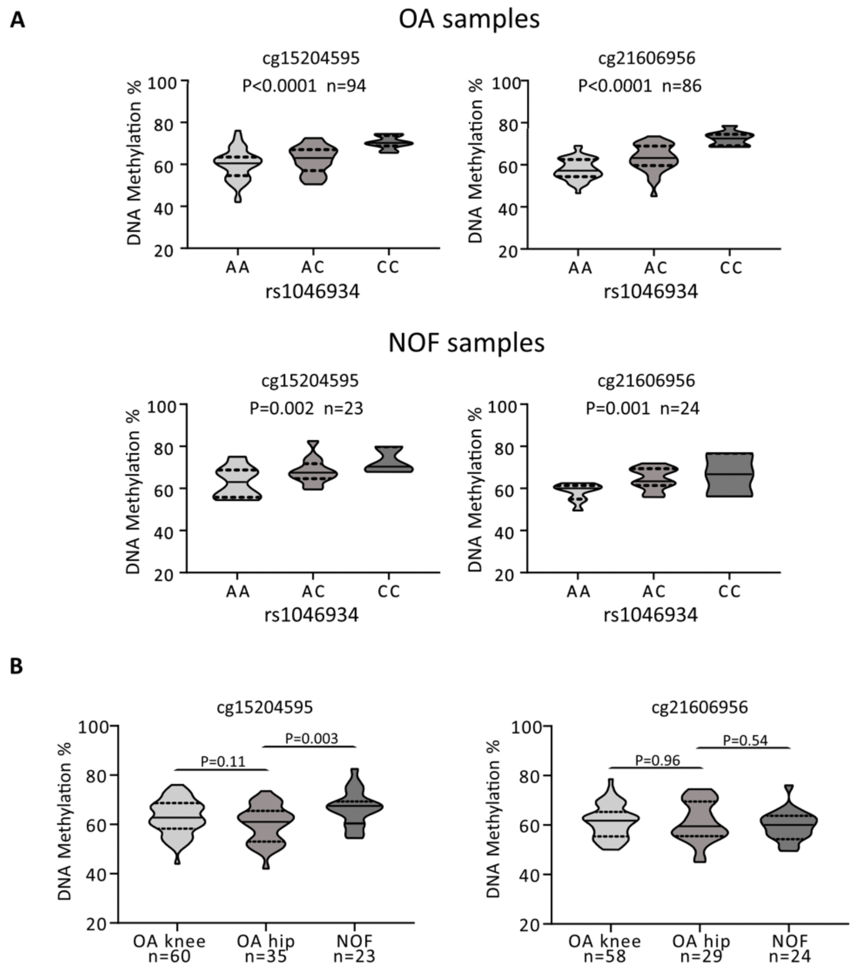

Supplementary Figure 1. Stratification of the arthroplasty methylation data. **A.** mQTL analysis with the replication data stratified into OA and femoral neck fracture (NOF). *P* values calculated by linear regression. **B.** Stratification of the methylation data by OA knee, OA hip and NOF irrespective of rs1046934 genotype. *P* values calculated by a Mann-Whitney U test. For A and B, the solid and dashed horizontal lines of the violin plots represent the median and interquartile range.

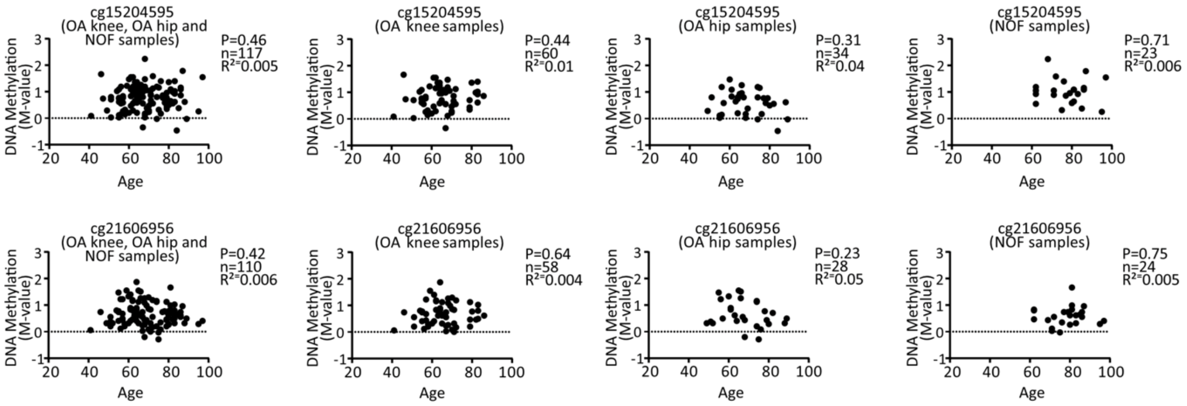

Supplementary Figure 2. Age versus methylation. Linear regression was used to test for association between age at surgery in years and DNAm levels at cg15204595 (top) and cg21606956 (bottom). Patients were studied combined (OA knee, OA hip and femoral neck fracture [NOF]) and following stratification. Each dot is data from one individual.

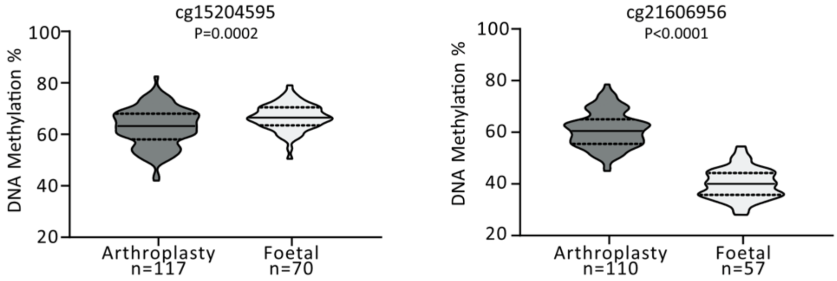

Supplementary Figure 3. CpG methylation levels in arthroplasty and fetal samples. Stratification of the arthroplasty and fetal methylation data irrespective of rs1046934 genotype. *P* values calculated by a Mann-Whitney U test. Solid and dashed horizontal lines of the violin plots represent the median and interquartile range.

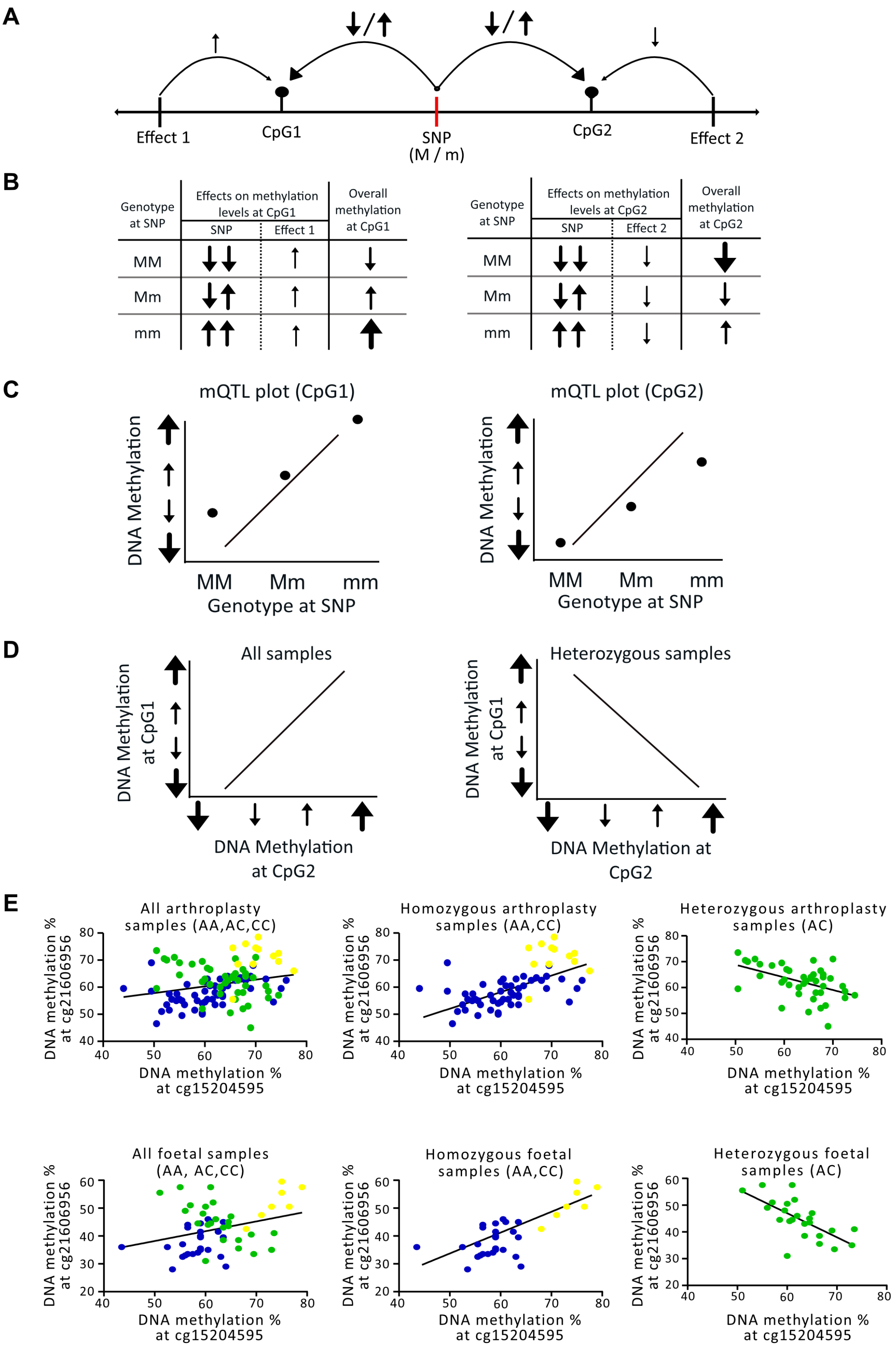

Supplementary Figure 4. Theoretical model to account for the opposing slopes of the *COLGALT2* meQTLs at cg15204595 and cg21606956. To be read in conjunction with Supplementary Text. **A.** Schematic diagram showing the factors affecting the methylation levels at CpG1 and CpG2 (marked by black circles). The SNP (M, major allele; m, minor allele)

is marked by a red line and Effect 1 and Effect 2 are marked with black lines. Arrows indicate the effect the different factors have on the methylation levels at the two CpG sites with the direction of the arrowhead indicating the direction of the effect (up = increased methylation; down = decreased methylation) and the thickness of the arrow corresponding to the strength of the effect (thin = weaker effect; thick = stronger effect). **B.** Table summarising the cumulative effect of the SNP and Effect 1 and Effect 2 on the methylation levels at CpG1 (left) and CpG2 (right) for individuals homozygous (MM or mm) and heterozygous (Mm) for the SNP. The effects are represented by arrows with the direction of the arrowhead indicating the direction of the effect (up = increased methylation; down = decreased methylation) and the thickness of the arrow corresponding to the strength of the effect (thin = weaker effect; thick = stronger effect). **C.** Predicted mQTL plots at CpG1 (left) and CpG2 (right) using the expected overall methylation levels presented in B. Methylation levels are represented on the y-axis by arrows with the direction of the arrowhead indicating the direction of the effect (up = increased methylation; down = decreased methylation) and the thickness of the arrow corresponding to the levels of methylation (thin = medium high/low levels; thick = very high/low levels). Genotype at the SNP is on the x-axis (MM, Mm or mm). The overall methylation levels expected for each genotype are represented by a dot and the mQTL direction is indicated by a line. **D.** Plots showing the expected overall methylation levels at CpG1 plotted against the expected overall methylation levels at CpG2 in all individuals (left) and in heterozygous individuals (right). Methylation levels are represented by arrows with the direction of the arrowhead indicating the direction of the effect (up = increased methylation; down = decreased methylation) and the thickness of the arrow corresponding to the levels of methylation (thin = medium high/low levels; thick = very high/low levels). The lines show the direction of the predicted associations. **E.** Plots showing the measured methylation levels at cg15204595 plotted against the measured methylation levels at cg21606956 in all arthroplasty (top left) and all fetal (bottom left) samples, in arthroplasty (top middle) and fetal (bottom middle) samples homozygous (AA and CC) for rs1046934, and in arthroplasty (top right) and fetal (bottom right) samples heterozygous (AC) for rs1046934. Each sample is represented by a dot with the colour of the dot corresponding to rs1046934 genotype (blue = AA, green = AC, yellow = CC). The trend lines show the direction of the associations.

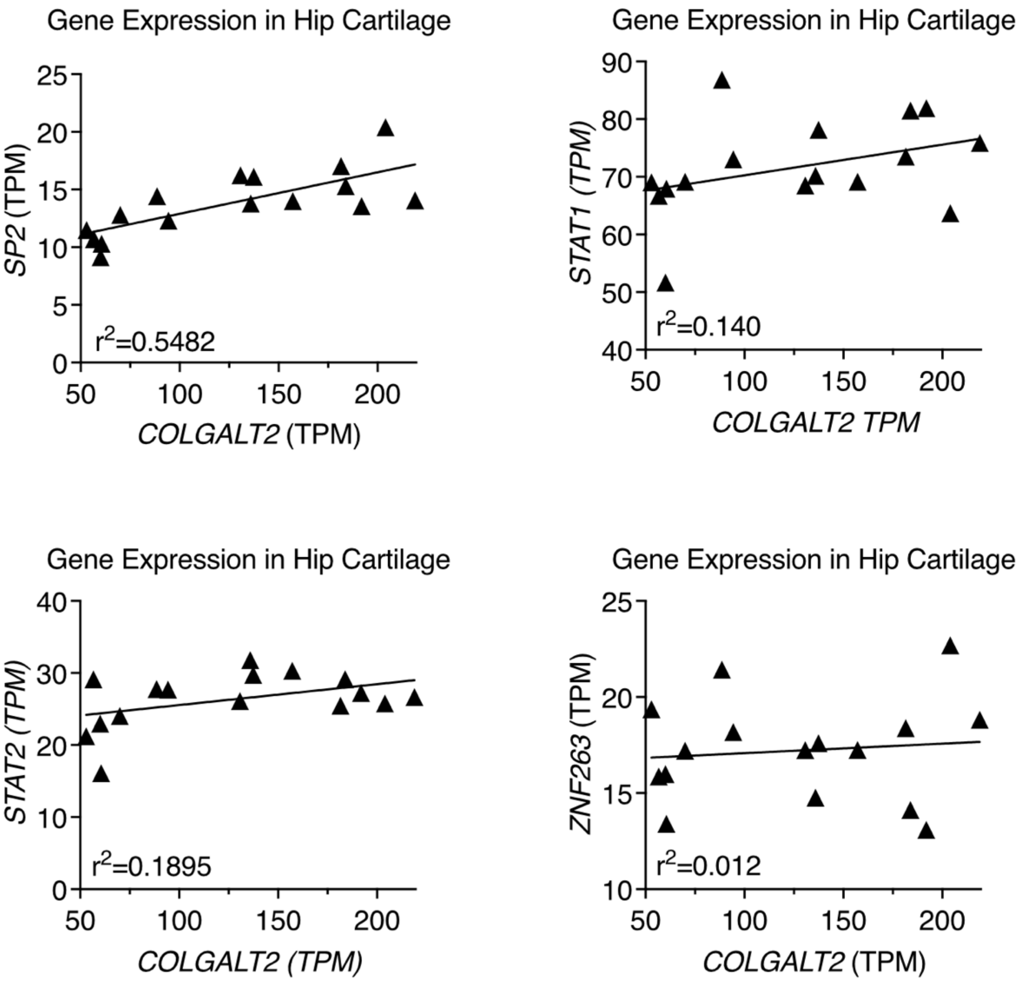

Supplementary Figure 5. Relationship between expression of *COLGALT2* and predicted functional transcription factors (TFs). Hip cartilage RNA-sequencing data from 16 arthroplasty patients (10 OA and 6 femoral neck fracture [NOF]). r^2 values calculated by linear regression. TPM, transcripts per million.

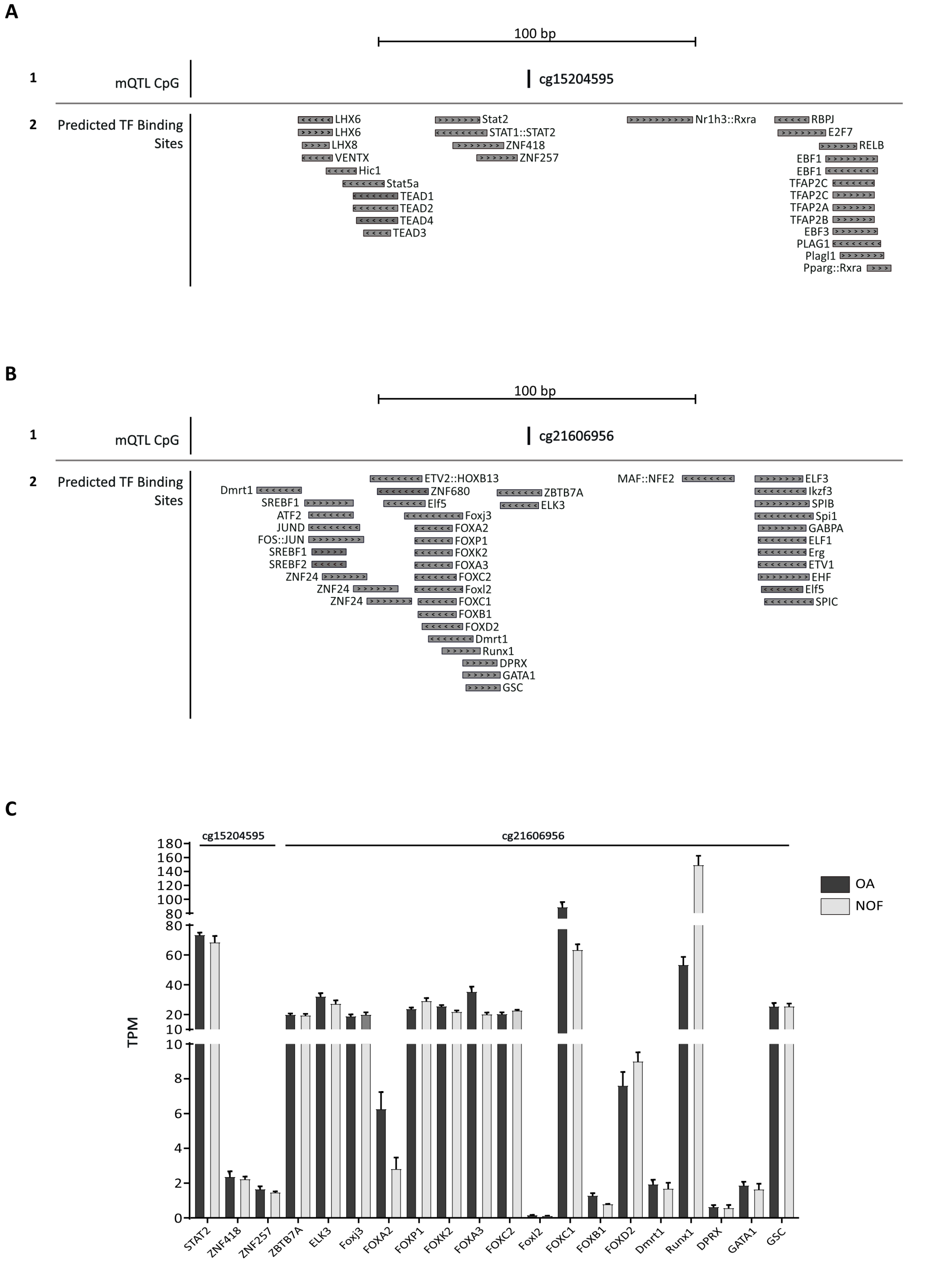

Supplementary Figure 6. Transcription factors (TFs) predicted to bind at or close to cg15204595 and cg21606956. TF binding sites within 200bp of cg15204595 (**A**) or 200bp of cg21606956 (**B**) as predicted by JASPAR, visualised in the UCSC Genome Browser (hg19). Sections 1 of **A** and **B** highlight the CpGs (black lines), sections 2 the positions of the TFs. The

TFs are marked by grey bars with the direction of the arrows within the boxes indicating the DNA strand the TF is predicted to bind to: arrows pointing to the left = antisense strand; arrows pointing to the right = sense strand. **C.** Expression levels (TPM, transcripts per million) of those TFs predicted to bind within 30bp of cg15204595 and cg21606956 in hip cartilage RNA-sequencing data from OA (n = 10, dark grey) and femoral neck fracture (NOF) (n = 6, light grey) patients. Bars show the mean ±SEM. Y-axis is a linear segmented scale with three segments.

Database	Data type	URL
Protein Data Bank	Macromolecular structures	https://www.rcsb.org
gnomAD	Impact on protein function of missense DNA variants	https://gnomad.broadinstitute.org
PolyPhen	Impact on protein function of missense DNA variants	http://genetics.bwh.harvard.edu/pph2/
Mutation Taster	Impact on protein function of missense DNA variants	https://www.mutationtaster.org/
ROADMAP	Chromatin state data	http://www.roadmapepigenomics.org
3D Genome Browser	Chromatin capture Hi-C	http://3dgenome.fsm.northwestern.edu/chic.php
UCSC Genome Browser	Integrated genomic datasets	https://genome.ucsc.edu
LDlink	SNP linkage disequilibrium measures	https://ldlink.nci.nih.gov/?tab=home
JASPAR	Transcription factor binding profiles	https://jaspar.genereg.net
SNP2TFBS	Effect of DNA variants on transcription factor binding sites	https://ccg.epfl.ch/snp2tfbs/
Gene Expression Omnibus (GEO)	Genomics data repository	https://www.ncbi.nlm.nih.gov/geo/

Supplementary Table 1. The public databases used in this study.

Sample ID	Sex	Joint	Age at surgery (years)	Disease
4809	Male	Knee	79	OA
4883	Female	Knee	68	OA
4971	Male	Knee	64	OA
5099	Female	Knee	57	OA
5103	Female	Knee	58	OA
5104	Female	Knee	67	OA
5137	Female	Hip	52	OA
5163	Male	Knee	55	OA
5164	Male	Knee	55	OA
5178	Female	Knee	83	OA
5188	Female	Knee	64	OA
5214	Male	Knee	57	OA
5764	Male	Hip	64	OA
5999	Female	Knee	79	OA
6135	Male	Knee	71	OA
6156	Female	Hip	67	OA
6184	Female	Knee	55	OA
6218	Female	Hip	79	OA
6224	Female	Knee	62	OA
6342	Female	Knee	62	OA
6359	Female	Knee	70	OA
6363	Female	Knee	68	OA
6378	Male	Knee	54	OA
6506	Female	Knee	59	OA
6770	Female	Knee	80	OA
6772	Male	Knee	71	OA
6778	Male	Knee	63	OA
6783	Female	Knee	55	OA
6784	Female	Knee	46	OA
6786	Male	Knee	82	OA
6787	Male	Knee	64	OA
6788	Male	Knee	66	OA
6803	Female	Knee	65	OA
6818	Female	Knee	62	OA
6852	Female	Knee	83	OA
6867	Male	Knee	86	OA
7007	Male	Hip	77	OA
7008	Female	Hip	75	OA
7015	Female	Hip	64	OA

7025	Female	Hip	76	OA
7029	Female	Hip	67	OA
7030	Male	Hip	63	OA
7034	Female	Hip	60	OA
7036	Female	Hip	88	OA
7037	Female	Hip	80	OA
7039	Female	Hip	68	OA
7040	Male	Hip	74	OA
7046	Female	Hip	56	OA
7047	Female	Hip	78	OA
7115	Female	Knee	83	OA
7116	Male	Knee	51	OA
7118	Female	Knee	47	OA
7120	Female	Knee	61	OA
7124	Male	Knee	64	OA
19	Male	Hip	66	OA
22	Male	Hip	48	OA
39	Female	Knee	72	OA
40	Female	Hip	84	OA
41	Male	Knee	51	OA
42	Female	Hip	71	OA
45	Male	Knee	83	OA
49	Male	Hip	68	OA
51	Female	Hip	74	OA
52	Female	Hip	74	OA
53	Female	Hip	55	OA
54	Male	Hip	52	OA
57	Male	Knee	63	OA
59	Female	Knee	60	OA
61	Male	Knee	67	OA
66	Female	Hip	56	OA
69	Male	Hip	57	OA
70	Male	Hip	75	OA
72	Male	Knee	76	OA
73	Female	Hip	46	OA
76	Female	Knee	65	OA
77	Female	Knee	71	OA
78	Male	Knee	58	OA
79	Male	Knee	65	OA
82	Female	Knee	67	OA
86	Female	Knee	56	OA
87	Female	Hip	51	OA

89	Female	Knee	67	OA
90	Female	Hip	60	OA
92	Female	Hip	55	OA
96	Female	Hip	61	OA
97	Male	Hip	65	OA
98	Male	Hip	49	OA
100	Female	Hip	74	OA
103	Female	Knee	70	OA
104	Female	Knee	70	OA
106	Male	Knee	79	OA
107	Female	Knee	41	OA
108	Male	Knee	72	OA
109	Female	Knee	65	OA
112	Male	Hip	61	OA
114	Female	Knee	57	OA
115	Male	Knee	69	OA
116	Male	Hip	89	OA
117	Male	Hip	82	OA
126	Female	Hip	66	OA
127	Male	Knee	61	OA
128	Male	Knee	64	OA
135	Female	Hip	69	OA
136	Female	Knee	68	OA
165	Female	Hip	64	OA
166	Female	Knee	62	OA
T023	Female	Hip	68	NOF
T117	Male	Hip	85	NOF
T151	Male	Hip	62	NOF
T167	Female	Hip	62	NOF
T168	Female	Hip	81	NOF
T172	Female	Hip	77	NOF
T174	Male	Hip	86	NOF
T177	Female	Hip	72	NOF
T178	Female	Hip	62	NOF
T179	Female	Hip	81	NOF
T184	Female	Hip	62	NOF
T191	Female	Hip	87	NOF
T192	Female	Hip	71	NOF
T193	Female	Hip	86	NOF
T195	Female	Hip	80	NOF
T196	Female	Hip	83	NOF
T204	Female	Hip	76	NOF

T244	Female	Hip	97	NOF
T245	Female	Hip	81	NOF
T246	Female	Hip	83	NOF
T005	Male	Hip	69	NOF
T007	Female	Hip	71	NOF
T150	Female	Hip	80	NOF
T152	Female	Hip	95	NOF
T154	Male	Hip	75	NOF
T158	Male	Hip	79	NOF

Supplementary Table 2. Details of the arthroplasty patient samples used in this study. OA, osteoarthritis; NOF, femoral neck fracture.

Sample ID	Sex	Limb/Joint	Developmental stage
14375	Male	Proximal and distal tibia	12pcw
14395	Female	Proximal and distal tibia	10pcw
14397	Female	Proximal and distal tibia and femur	15pcw
14423	Male	Proximal and distal tibia	12pcw
14429	Female	Proximal and distal tibia	14pcw
14451	Male	Proximal and distal femur	9pcw
14453	Female	Proximal and distal femur	12pcw
14460	Female	Proximal and distal tibia	9pcw
14464	Male	Proximal and distal tibia	16pcw
14466	Male	Proximal and distal tibia	12pcw
14467	Male	Proximal and distal tibia	10pcw
14475	Male	Proximal and distal tibia	16pcw
14408	Female	Proximal and distal tibia	14pcw
14501	Male	Proximal and distal tibia	16pcw
14513	Female	Proximal and distal tibia and femur	12pcw
14563	Male	Proximal and distal tibia	14pcw
14586	Female	Proximal and distal tibia and femur	13pcw
14604	Female	Proximal and distal tibia	14pcw
14471	Female	Proximal and distal tibia and femur	9pcw
14555	Male	Proximal and distal tibia and femur	9pcw
14453	Female	Proximal and distal femur	12pcw
14562	Female	Proximal and distal tibia	9pcw
14580	Male	Proximal and distal tibia and femur	9pcw
14600	Male	Proximal and distal tibia	8pcw
14576	Male	Proximal and distal tibia and femur	8pcw
14544	Male	Proximal and distal tibia	13pcw
14684	Female	Proximal and distal femur	14pcw
14606	Female	Proximal and distal tibia and femur	9pcw
14617	Female	Proximal and distal tibia and femur	13pcw
14377	Male	Proximal and distal tibia	14pcw
14378	Male	Proximal and distal tibia	14pcw
14392	Male	Proximal and distal tibia	9pcw
14393	Male	Proximal and distal tibia	14pcw
14408	Female	Proximal and distal tibia	14pcw
14472	Male	Proximal and distal tibia	12pcw
14492	Female	Proximal and distal tibia	12pcw
14510	Female	Proximal and distal tibia	14pcw
14511	Female	Proximal and distal tibia	11pcw
14512	Male	Proximal and distal tibia	10pcw
14516	Male	Proximal and distal tibia	15pcw

14521	Male	Proximal and distal tibia	12pcw
14522	Female	Proximal and distal tibia	15pcw
14523	Female	Proximal and distal tibia	15pcw
14524	Female	Proximal and distal tibia	15pcw
14525	Male	Proximal and distal tibia	10pcw
14532	Female	Proximal and distal tibia	11pcw
14541	Female	Proximal and distal tibia	11pcw
14577	Female	Proximal and distal tibia and femur	11pcw
14597	Male	Proximal and distal tibia	11pcw
14601	Male	Proximal and distal tibia and femur	10pcw
14603	Female	Proximal and distal tibia and femur	11pcw
14619	Male	Proximal and distal tibia and femur	16pcw
14628	Female	Proximal and distal tibia and femur	17pcw
14681	Female	Proximal and distal tibia and femur	11pcw
14688	Male	Proximal and distal tibia and femur	11pcw
14689	Male	Proximal and distal femur	10pcw
14703	Female	Proximal and distal tibia and femur	12pcw
14713	Female	Proximal and distal tibia and femur	16pcw
14715	Male	Proximal and distal tibia and femur	12pcw
14716	Male	Proximal and distal tibia and femur	10pcw
14717	Female	Proximal and distal tibia	14pcw
14720	Female	Proximal and distal tibia and femur	15pcw
14721	Female	Proximal and distal tibia and femur	10pcw
14722	Male	Proximal and distal tibia and femur	17pcw
14727	Male	Proximal and distal tibia	12pcw
14728	Male	Proximal and distal tibia and femur	16pcw
14729	Male	Proximal and distal tibia and femur	12pcw
14738	Male	Proximal and distal tibia and femur	15pcw
14746	Female	Proximal and distal tibia and femur	16pcw
14748	Female	Proximal and distal tibia and femur	10pcw
14749	Male	Proximal and distal tibia and femur	13pcw
14782	Male	Proximal and distal tibia and femur	10pcw
14797	Male	Proximal and distal tibia and femur	12pcw
14831	Female	Proximal and distal tibia and femur	9pcw
14835	Female	Proximal and distal tibia and femur	12pcw
14863	Male	Proximal and distal tibia and femur	10pcw
14881	Female	Proximal and distal tibia and femur	10pcw

Supplementary Table 3. Details of the fetal cartilage samples used in this study. PCW, post conceptional weeks.

Experiment	SNP/CpG	Position of SNP/CpG (hg19)	Oligonucleotide		
			Forward (5'-3')	Reverse (5'-3')	Sequencing (5'-3')
Genotyping	rs1046934	chr1:184023529	TTAGATATAGGAGATGCCACCCA	CAGGTAAACCAAGAACGCTACATA	AGGAGATGCCACCCA
Genotyping and AEI	rs114661926	chr1:184006549	GAGAGGAGCAGTAGCGACCAGG	AGCAAGGGGCTGCGAGGG	TGAGGGCGGCGGCGG
	rs2274432	chr1:184020945	GGGAAGCGGAACCCACAG	CCCTGAGGACGCCTGGAT	TGTTCGCGGCTTTGG
Methylation	cg15204595	chr1:184003184	TGTGGTTAGATTTAGAGGAAGTTGA	AACACTCTAATAAAAAATAATACCTCAATT	ATGAGAAATTAGAGGTAAGT
	cg21606956	chr1:184211935	TTTTGTATTTATTTTGTGGTTAATTTTTG	CAATAACTCTATACTATAATAAAATCCTCT	TGTGGTTAATTTTTGGTTT

Supplementary Table 4. Sequence of oligonucleotide primers used for genotyping, allelic expression imbalance (AEI) analysis, and methylation analysis.

Gene	SNP	Genome position (hg19)	Location in gene	Alleles (MAF in EUR)	OA-risk allele	Pairwise r2 with rs1046934 in EUR
COLGALT2	rs114661926	chr1:184006549	5'UTR	C>G (0.304)	C	0.79
TSEN15	rs2274432	chr1:184020945	Exonic	G>A (0.327)	G	1

Supplementary Table 5. Proxy transcript SNPs used for AEI analyses. MAF, minor allele frequency; EUR, European ancestry cohorts; UTR, untranslated region.

Experiment	Oligonucleotide/gRNA name	Sequence (5'-3')
Lucia - cg15204595	Lucia595 FWD	ATGCATTCAGTTTGGCATCTTGGAATGT
	Lucia595 REV	ACTAGTGATAATAGGGCATCAACCAGAGT
Lucia - cg21606956	Lucia956 FWD	ATGCATCAAAGTTCTGGGTAGCTCACA
	Lucia956 REV	ACTAGTGAATCTCTTTGGAAGTGCTGACT
Demethylation/Methylation of cg15204595	gRNA1 FWD	CACCGTGGAATGTTGCATAGAGCTT
	gRNA1 REV	AAACAAGCTCTATGCAACATTCCAC
Demethylation/Methylation of cg21606956	gRNA2 FWD	CACCGGTTATTGTGGTCAGATTCAG
	gRNA2 REV	AAACCTGAATCTGACCACAATAACC

Supplementary Table 6. Sequences of the oligonucleotide primers used for cloning the regions containing cg15204595 and cg21606959 into the Lucia CpG-free-promoter vector, and of the gRNAs used for the targeted demethylation/methylation of cg15204595 and cg21606959. Sequences in red for the Lucia oligonucleotides are restriction enzyme sites used for cloning - ATGCAT, NsiI; ACTAGT, SpeI. Sequences in red for the gRNA oligonucleotides are to facilitate cloning.

Gene	Assay
COLGALT2	Hs.PT.58.19198739
TSEN15	Hs.PT.58.24776232

Supplementary Table 7. The pre-designed IDT RT-qPCR assays used to quantify gene expression.

CpG	Nominal P value	Slope	CpG.chr	CpG.pos	CpG.strand	CpG.Island	CpG.Group	CpG.GeneSymbol
cg21606956	0.00238879	0.158917453	chr1	184211934	-	OpenSea		
cg15204595	0.005077495	0.255571328	chr1	184003184	+	N_Shelf	Body	*COLGALT2*
cg01436608	0.04128075	0.178202059	chr1	184005534	-	Island	Body	*COLGALT2*
cg22340762	0.051489372	-0.177768504	chr1	183823538	+	OpenSea	Body	*RGL1*
cg17002156	0.054070936	0.232924321	chr1	183995878	+	OpenSea	Body	*COLGALT2*
cg21404028	0.10836635	0.092775989	chr1	184020687	-	N_Shore	TSS200;TSS200;TSS200	*TSEN15;TSEN15;TSEN15*
cg12222949	0.1130119	0.16041609	chr1	184005360	+	Island	Body	*COLGALT2*
cg15111486	0.133341488	-0.104192865	chr1	184195211	-	OpenSea		
cg05777716	0.144389699	-0.080063479	chr1	184006285	-	Island	1stExon	*COLGALT2*
cg13951632	0.184928856	0.120100661	chr1	183897368	-	OpenSea	3'UTR	*RGL1*
cg15569801	0.187897516	0.066928549	chr1	184197096	-	OpenSea		
cg27177158	0.194193974	0.100123681	chr1	184121585	-	OpenSea		
cg02314308	0.194957334	0.110305009	chr1	184006972	-	S_Shore	TSS200	*COLGALT2*
cg27444414	0.230101787	0.092115213	chr1	184122874	-	OpenSea		
cg10312498	0.240591977	-0.109116001	chr1	183957982	-	OpenSea	Body	*COLGALT2*
cg16655084	0.243080607	0.081155105	chr1	184005896	+	Island	Body	*COLGALT2*
cg12136256	0.248654028	-0.136178579	chr1	183952796	+	OpenSea	Body	*COLGALT2*
cg14773259	0.292969986	0.085088592	chr1	184021034	-	Island	Body;Body;Body	*TSEN15;TSEN15;TSEN15*
cg22400420	0.304191771	-0.088312676	chr1	183851242	-	OpenSea	Body	*RGL1*
cg18131582	0.363285827	0.104087735	chr1	183912305	-	OpenSea	Body	*COLGALT2*
cg00464025	0.381248495	-0.040631933	chr1	183907542	-	OpenSea	3'UTR	*COLGALT2*
cg19101018	0.385337956	-0.063402477	chr1	183925532	-	OpenSea	Body	*COLGALT2*
cg21417627	0.447531032	-0.067376864	chr1	184008235	-	S_Shore	TSS1500	*COLGALT2*
cg14313310	0.485865459	0.055906007	chr1	184006816	-	S_Shore	5'UTR;1stExon	*COLGALT2;COLGALT2*
cg09940311	0.496887744	-0.110077477	chr1	183901666	-	OpenSea		
cg06849459	0.514610056	-0.04667026	chr1	183891439	-	OpenSea	Body	*RGL1*
cg01068808	0.530912366	-0.075189524	chr1	184007101	-	S_Shore	TSS1500	*COLGALT2*
cg18073970	0.542309516	-0.057603771	chr1	184006765	+	Island	5'UTR;1stExon	*COLGALT2;COLGALT2*
cg09684066	0.558049453	0.031355578	chr1	183891565	+	OpenSea	Body	*RGL1*
cg25605307	0.560179747	-0.0498599	chr1	183965312	-	OpenSea	Body	*COLGALT2*
cg11678039	0.580064206	0.041394928	chr1	183841259	-	OpenSea	Body	*RGL1*
cg16716196	0.595667485	0.049572938	chr1	184195068	-	OpenSea		
cg03467001	0.604461199	-0.063457988	chr1	183857649	-	OpenSea	Body	*RGL1*
cg03943177	0.611155155	0.052335755	chr1	184021360	+	S_Shore	Body;Body;Body	*TSEN15;TSEN15;TSEN15*
cg06915270	0.642327728	0.027943555	chr1	183891360	+	OpenSea	Body	*RGL1*
cg17357479	0.658357979	-0.037040788	chr1	184005717	-	Island	Body	*COLGALT2*
cg10807943	0.689234253	-0.020570128	chr1	184021131	+	S_Shore	Body;Body;Body	*TSEN15;TSEN15;TSEN15*
cg12702671	0.711635427	0.031402346	chr1	183885210	-	OpenSea	Body	*RGL1*
cg20942099	0.7233262	-0.023790576	chr1	184007803	+	S_Shore	TSS1500	*COLGALT2*
cg09013655	0.754989126	-0.032897006	chr1	184005063	-	N_Shore	Body	*COLGALT2*
cg12792264	0.765268544	-0.035320275	chr1	183971636	+	OpenSea	Body	*COLGALT2*
cg00044463	0.766522434	0.02378764	chr1	184006933	+	S_Shore	TSS200	*COLGALT2*
cg24721630	0.769637772	0.027460413	chr1	184006216	+	Island	Body	*COLGALT2*
cg02890642	0.78153886	-0.021560653	chr1	184017798	-	N_Shelf		
cg27291258	0.784525638	-0.043489618	chr1	184173289	+	OpenSea		
cg12758231	0.787779993	0.019910919	chr1	184020711	+	N_Shore	TSS200;TSS200;TSS200	*TSEN15;TSEN15;TSEN15*
cg15359501	0.792639164	0.015547072	chr1	184006496	-	Island	5'UTR;1stExon	*COLGALT2;COLGALT2*
cg26429856	0.798737947	0.013574529	chr1	184005990	+	Island	Body	*COLGALT2*
cg18189236	0.821703244	-0.025694924	chr1	184009497	-	S_Shelf		
cg05816006	0.839369641	-0.018867477	chr1	184042579	+	OpenSea	3'UTR;3'UTR;Body	*TSEN15;TSEN15;TSEN15*
cg11387897	0.849670158	0.012868174	chr1	183914188	-	OpenSea	Body	*COLGALT2*
cg12631766	0.887366794	-0.015505912	chr1	184025029	-	S_Shelf	Body;Body;Body	*TSEN15;TSEN15;TSEN15*
cg11346030	0.915841776	0.009511507	chr1	184090950	-	OpenSea		
cg06379531	0.921982985	-0.00580673	chr1	184020410	-	N_Shore	TSS1500;TSS1500;TSS1500	*TSEN15;TSEN15;TSEN15*
cg23040305	0.96965166	0.004039352	chr1	183846243	-	OpenSea	Body	*RGL1*
cg15521745	0.983100265	0.001355847	chr1	184133404	-	OpenSea		
ch.1.182359526R	0.987854652	0.001960897	chr1	184092903	+	OpenSea		
cg20271396	0.99541804	-0.000473252	chr1	184020713	+	N_Shore	TSS200;TSS200;TSS200	*TSEN15;TSEN15;TSEN15*

Supplementary Table 8. rs1046934 mQTL analysis of CpGs located 200kb upstream and downstream of the SNP. CpGs are ranked by *P* value. CpGs with $P < 0.05$ are highlighted.

SNP	Chromosome	Start (hg19)	End (hg19)	Alleles	MAF	Distance	D'	r2	TF Binding?	TF1 TPM	TF2 TPM	ROADMAP
rs74767794	chr1	184006128	184006129	(A/G)	0.3002	17401	0.9557	0.8063	Yes	SP2 (13.9)		Promoter (COLGALT2)
rs3814333	chr1	184007119	184007120	(C/T)	0.3002	16410	0.9557	0.8063	No			Promoter (COLGALT2)
rs1926874	chr1	184009788	184009789	(C/T)	0.325	13741	0.9773	0.9465	No			Quiescent (Intergenic)
rs5779197	chr1	184009826	184009827	(-/AA)	0.3241	13703	0.9772	0.9421	Yes	STAT1 (26.3)	STAT2 (71.7)	Quiescent (Intergenic)
rs10494574	chr1	184010727	184010728	(C/A)	0.332	12802	0.9681	0.9165	No			Quiescent (Intergenic)
rs4650650	chr1	184012841	184012842	(A/G)	0.325	10688	0.9773	0.9465	Yes	SP2 (13.9)	ZNF263 (17.2)	Quiescent (Intergenic)
rs12029527	chr1	184013318	184013319	(C/A)	0.325	10211	0.9773	0.9465	No			Quiescent (Intergenic)
rs12047271	chr1	184013491	184013492	(T/C)	0.325	10038	0.9773	0.9465	No			Quiescent (Intergenic)
rs12022430	chr1	184013495	184013496	(A/G)	0.325	10034	0.9773	0.9465	Yes	FEV (0.1)		Quiescent (Intergenic)
rs12044334	chr1	184013831	184013832	(G/C)	0.326	9698	0.9728	0.9421	No			Quiescent (Intergenic)
rs1327124	chr1	184014199	184014200	(A/G)	0.329	9330	0.9683	0.9292	No			Quiescent (Intergenic)
rs1327123	chr1	184014593	184014594	(G/C)	0.327	8936	0.991	0.982	Yes	Pax4 (0.4)	ZNF354C (1.7)	Quiescent (Intergenic)
rs10797937	chr1	184017131	184017132	(A/G)	0.326	6398	0.9955	0.9865	No			Quiescent (Intergenic)
rs1926872	chr1	184018475	184018476	(T/C)	0.326	5054	0.9955	0.9865	Yes	Crx (6.2)		Quiescent (Intergenic)
rs71694372	chr1	184018973	184018974	(TCTT/-)	0.326	4556	0.9955	0.9865	No			Quiescent (Intergenic)
rs10797938	chr1	184019954	184019955	(C/A)	0.326	3575	0.9955	0.9865	No			Enhancer
rs2274432	chr1	184020945	184020946	(G/A)	0.327	2584	1	1	No			Promoter (Exon 1, TSEN15)
rs1046934	chr1	184023529	184023530	(A/C)	0.303	n/a	1	1	No			Transcribed (Exon 2, TSEN15)
rs112621660	chr1	184029915	184029916	(AGTT/-)	0.327	6386	1	1	No			Transcribed (Intronic, TSEN15)
rs1952256	chr1	184035116	184035117	(A/G)	0.327	11587	1	1	No			Transcribed (Intronic, TSEN15)
rs3032594	chr1	184035420	184035421	(TATT/-)	0.327	11891	1	1	No			Transcribed (Intronic, TSEN15)

Supplementary Table 9. SNPs in the LD block marked by rs1046934. MAF, minor allele frequency in European ancestry cohorts (EUR); Distance, base pair distance from rs1046934; TF binding, SNP predicted to impact transcription factor binding based on position weight score (PWS) in the SNP2TFBS database; TF1, TF2, transcription factors with predicted altered binding introduced by the SNP, and their mean expression value in arthroplasty hip cartilage expressed as transcripts per million (TPM); ROADMAP, chromatin state data from ROADMAP 15 state model in MSC-derived chondrocytes (E049).

Supplementary text

Accounting for the observation that the *COLGALT2* meQTLS have opposing slopes.

To be read in conjunction with Supplementary Figure 4.

The OA risk-conferring allele A of rs1046934 is associated with lower methylation levels at cg15204595 and cg21606956 compared to the non-risk allele C in both arthroplasty (Figure 4A) and fetal (Figure 5B) samples. Allele A is also associated with higher *COLGALT2* expression, as evidenced by the AEI analyses, in both tissue types (Figure 2A and Figure 5A). Methylation-expression (meQTL) plots show associations between methylation levels at both CpGs and *COLGALT2* AEI ratios (Figure 4B and Figure 5C). However, while higher methylation levels at cg15204595 are associated with lower allelic ratios, the opposite is observed at cg21606956, with higher methylation levels corresponding to higher allelic ratios. This is true for both arthroplasty (Figure 4B) and fetal (Figure 5C) samples.

This can be explained if the methylation levels at the two CpGs are influenced by factors other than rs1046934 genotype.

In a theoretical model where a regulatory SNP affects methylation levels at two CpG sites (CpG1 and CpG2), with its major allele (M) associated with low methylation and its minor allele (m) with high methylation at both CpG1 and CpG2, individuals homozygous for the major allele (MM) will have low methylation levels, individuals homozygous for the minor allele (mm) will have high methylation levels, and heterozygous individuals will have intermediate methylation levels at both CpGs (Supplementary Figure 4A and 4B). Assume two additional factors affect the methylation levels at CpG1 and CpG2 in this theoretical model: Effect 1, which has a constant effect on the methylation at CpG1 leading to high levels of methylation at the CpG, and Effect 2, which has a constant effect on the methylation at CpG2 leading to low levels of methylation at the CpG (Supplementary Figure 4A and 4B). If these two hypothetical effects are weaker than the effect the SNP has on the methylation at CpG1 and CpG2, they will be masked in individuals homozygous at the SNP (MM or mm). However, in heterozygous individuals (Mm), they will not be masked: Effect 1 will complement the effect of the minor allele (m) of the SNP and the overall methylation levels at CpG1 will be higher than the expected mean but lower than (mm) homozygotes (Supplementary Figure 4B, left); Effect 2 will complement the effect of the major allele (M) of the SNP and the overall methylation levels at CpG2 will be lower than the expected mean but higher than (MM) homozygotes (Supplementary Figure 4B, right).

If both the effects of the SNP and Effect 1 or Effect 2 (for CpG1 and CpG2, respectively) are taken into consideration, the expected mQTL plots for CpG1 and CpG2 will show a similar trend (Supplementary Figure 4C). If the methylation levels at CpG1 are plotted against the levels at CpG2 for all individuals irrespective of SNP genotype, low levels of methylation at one CpG will correspond to low levels at the other (Supplementary Figure 4D, left). When the same plot is created for heterozygous individuals, however, the trend is reversed and low methylation levels at one CpG will correspond to higher levels at the other (Supplementary Figure 4D, right). This observation is expected since the weaker effects of Effect 1 and Effect 2 complement the effects of the minor and the major alleles (respectively) of the SNP, with the SNP alleles have opposing effects on methylation levels.

The theoretical scenarios described here are consistent with our actual observations in arthroplasty and fetal samples. When methylation levels at cg15204595 and cg21606956

are plotted against genotype at rs1046934 they show a similar trend in both arthroplasty (Figure 4A) and fetal (Figure 5B) samples. When methylation levels at cg15204595 are plotted against the levels at cg21606956 for all arthroplasty (Supplementary Figure 4E, top left) and all fetal (Supplementary Figure 4E, bottom left) samples, low methylation levels at one CpG associate with low methylation levels at the other. This is particularly striking when only rs1046934 homozygotes (AA and CC) are plotted (Supplementary Figure 4E, top middle [arthroplasty] and bottom middle [fetal]). However, in heterozygous (AC) samples (Supplementary Figure 4E, top right [arthroplasty] and bottom right [fetal]), low methylation at one CpG associates with higher methylation at the other.

The *COLGALT2* meQTL plots in Figure 4B and Figure 5C show methylation levels at cg15204595 and cg21606956 plotted against AEI ratios in arthroplasty and fetal samples. The AEI analysis is carried out in heterozygous individuals in which lower methylation levels at cg15204595 correspond to higher methylation levels at cg21606956. This leads to the meQTL plots having opposing slopes.
